# The plasmepsin-piperaquine paradox persists in *Plasmodium falciparum*

**DOI:** 10.1371/journal.ppat.1012779

**Published:** 2025-07-28

**Authors:** Breanna Walsh, Robert L. Summers, Gabriel W. Rangel, Laura M. Hagenah, Sachel Mok, Manuel Llinás, David A. Fidock, Dyann F. Wirth, Selina Bopp

**Affiliations:** 1 Department of Immunology and Infectious Diseases, Harvard T.H. Chan School of Public Health, Boston, Massachusetts, United States of America; 2 Department of Biochemistry and Molecular Biology and The Huck Center for Malaria Research, The Pennsylvania State University, University Park, Pennsylvania, United States of America; 3 Center for Malaria Therapeutics and Antimicrobial Resistance, Division of Infectious Diseases, Department of Medicine, Columbia University Irving Medical Center, New York, New York, United States of America; 4 Department of Chemistry, The Pennsylvania State University, University Park, Pennsylvania, United States of America; 5 Department of Microbiology and Immunology, Columbia University Irving Medical Center, New York, New York, United States of America; 6 Infectious Disease and Microbiome Program, The Broad Institute, Cambridge, Massachusetts, United States of America; University of Geneva Faculty of Medicine: Universite de Geneve Faculte de Medecine, SWITZERLAND

## Abstract

Malaria remains a pressing global health challenge, with rising drug resistance threatening current treatment strategies. Partial resistance to dihydroartemisinin-piperaquine (DHA-PPQ) has emerged in Southeast Asia, particularly in *Plasmodium falciparum* strains from Cambodia. While artemisinin partial resistance is associated with mutations in *kelch13*, reduced PPQ sensitivity has been linked to increased copy numbers of the aspartic protease genes *plasmepsin II* and *III* and mutations in the chloroquine resistance transporter. In this study, we demonstrate the effective use of CRISPR-Cas9 technology to generate single knockouts (KO) of *plasmepsin II* and *plasmepsin III*, as well as a double KO of both genes, in two isogenic Cambodian parasites with varying numbers of *plasmepsin* gene copies. The deletion of *plasmepsin II* and/or *III* increased parasite sensitivity to PPQ. We explored several hypotheses to understand how an increased *plasmepsin* gene copy number might influence parasite survival under high PPQ pressure. Our findings indicate that protease inhibitors have a minimal impact on parasite susceptibility to PPQ. Additionally, parasites with higher *plasmepsin* gene copy numbers did not exhibit significantly increased hemoglobin digestion, differences in peptide composition, nor did they produce different amounts of free heme following PPQ treatment compared to wildtype (single copy) parasites. Interestingly, hemoglobin digestion was slowed in parasites with *plasmepsin II* deletions. We also found that culturing parasites with different *plasmepsin II* and *III* copies in amino acid-limited media had little impact on parasite sensitivity to high-dose PPQ. By treating parasites with modulators of digestive vacuole (DV) homeostasis, we found that changes in DV pH potentially affect their response to PPQ. Our research highlights the crucial role of increased *plasmepsin II and III* gene copy numbers in modulating response to PPQ and begins to uncover the molecular and physiological mechanisms underlying the contribution of *plasmepsin II* and *III* amplification to PPQ resistance in Cambodian parasites.

## Author summary

Global malaria control has plateaued, with drug-resistant *Plasmodium falciparum* posing a significant challenge. Artemisinin-based combination therapies (ACTs) are becoming less effective, especially in Southeast Asia, where resistance to dihydroartemisinin-piperaquine (DHA-PPQ) resulted in high rates of treatment failures, notably in Cambodia. Genome-wide association studies link artemisinin partial resistance to *kelch13* mutations, while decreased PPQ sensitivity is associated with higher *plasmepsin II* and *III* gene copies and mutations in the chloroquine resistance transporter *Pf*CRT.

We previously showed a connection between increased *plasmepsin II* and *III* gene copy number and reduced PPQ sensitivity. In this study, we try to understand the biological role of the Plasmepsins in PPQ sensitivity. Therefore, we knocked out *plasmepsin II* and/or *III* genes in Cambodian strains using CRISPR-Cas9, and found increased PPQ sensitivity, confirming these genes’ roles in resistance. Plasmepsins are proteases that participate in the hemoglobin degradation cascade in the digestive vacuole of the parasites. However, we detected no evidence that hemoglobin digestion was significantly altered in parasites with increased *plasmepsin II* and *III* copy numbers. Altogether, this work elaborates on our current understanding of how *plasmepsin II* and *III* amplifications contribute to PPQ resistance and highlights the need for further research into PPQ resistance mechanisms.

## Introduction

Despite remarkable strides toward global malaria control since the early 2000s, progress in reducing the total number of malaria cases has plateaued [1]. Drug resistance in *Plasmodium falciparum* parasites, particularly to first- and second-line artemisinin (ART)-based combination therapies (ACTs), remains a major challenge to malaria elimination. In regions of Southeast Asia, resistance to both potent but short-lived ART-derived compounds and long-lasting partner drugs is emerging and rapidly spreading [2]. Widespread parasite resistance to dihydroartemisinin-piperaquine (DHA-PPQ) has resulted in clinical treatment failures throughout western Cambodia, where DHA-PPQ was adopted in 2008 [3–7]. PPQ resistance is marked by parasite recrudescence 42 days post-treatment [3]. The spread of PPQ-resistant parasites in Southeast Asia has significantly shortened the usable lifespan of DHA-PPQ, resulting in Cambodia’s 2016 reversal to artesunate-mefloquine at a first-line antimalarial [8].

There is a well-established association between mutations in the propeller region of *kelch13* and ART treatment failure in *P. falciparum* parasites, including those collected from patient samples in Southeast Asia by the Tracking Resistance to Artemisinin Collaboration (TRAC) used in this study [9–14]. GWAS of Cambodian *P. falciparum* isolates have revealed that decreased PPQ sensitivity is associated with increased copy numbers of the genes coding for the aspartic proteases Plasmepsin II and III in combination with a single copy of the multidrug resistance-1 (*pfmdr1*) gene [15,16]. *In vitro* studies in the laboratory strain 3D7 identified a slight sensitization to PPQ when *plasmepsin II* and *III* were deleted [17], but no susceptibility change was observed when *plasmepsin II* and *III* were overexpressed [18]. Furthermore, SNPs in the chloroquine resistance transporter (*pfcrt*) gene have also been associated with PPQ resistance [5,15,16,19]. Mutations in *pfcrt* have been shown to confer PPQ resistance when introduced into the Dd2 laboratory strain [20–23]. *In vitro* PPQ resistance has also been observed in South America, where field isolates from Guiana and Suriname carried a *Pf*CRT C350R variant in combination with *plasmepsin II* and *III* amplifications [24]. To date, parasites with mutations in *kelch13*, increased *plasmepsin* copy numbers, and *pfcrt* mutations are now almost fixed in Southeast Asia [25–28], the treatment failure rate increased up to 70% in Western Cambodia [2], and surveillance of these markers is now underway in Africa and South America.

Despite the identification of promising molecular markers, the characterization of PPQ resistance in parasite field isolates has proved challenging. When subjected to a standard drug susceptibility assay, PPQ-resistant parasites yield bimodal dose-response curves, with increased parasite survival at high PPQ concentrations. These bimodal curves cannot be described using traditional non-linear regression analysis and yield non-interpretable EC_50_ values when a curve fit is forced [4,7]. As a result, many practitioners rely on estimates of the EC_90_ instead. A PPQ survival assay (PSA) was developed, wherein parasite survival after 48 h of a single PPQ treatment (200 nM) is compared to that of parasites cultured in vehicle control [5]. However, PSA analysis is labor and time intensive. To better describe the bimodal response, we increased the concentration range of PPQ and utilized the area under the curve (AUC) of the high-dose peak to quantify the PPQ response [29]. Culture-adapted TRAC isolates showed a correlation between increased *plasmepsin II* and **plasmepsin* III* copy numbers and AUC. A panel of clonal isogenic lines, all with single copies of *pfmdr1* and identical *pfcrt* loci, showed decreased sensitivity to PPQ with increasing copy numbers of *plasmepsin II* and *plasmepsin III*, implicating these copy number variations (CNVs) as potential drivers of PPQ resistance [29]. Analysis of *plasmepsin II* and *plasmepsin III* KOs in the relevant genetic background of Cambodian isolates is key to further understanding the role of genetic background in modulating Plasmepsin II and III action under PPQ pressure.

Plasmepsin II and III, along with Plasmepsin I and IV, are aspartic proteases that participate in the hemoglobin degradation cascade in the digestive vacuole (DV) [30–34]. RNAseq data suggests that Plasmepsin III is maximally transcribed in late ring stages while Plasmepsin II peaks later during the early trophozoite stage, which might reflect a greater role for Plasmepsin III earlier in the intra-erythrocytic developmental cycle [35]. Targeted genetic disruptions of the *plasmepsin* genes - either individually or in combination - yield viable parasites lacking dramatic changes in morphology or growth, suggesting functional redundancy between these aspartic proteases and other proteases in the DV [34,36]*.* The digestion of hemoglobin crucially provides a source of amino acids for parasites; however, the breakdown of hemoglobin releases toxic free heme, which aggregates as inert, crystalline hemozoin within the DV [37–39]. As an aminoquinoline, PPQ is thought to impede the degradation of hemoglobin within the parasite DV, leading to a build-up of toxic free heme and parasite death [21,39]. However, the biological mode of PPQ action is not well understood.

Here, we show successful CRISPR-Cas9-mediated *plasmepsin II* single KO, *plasmepsin III* single KO, and *plasmepsin II* and *plasmepsin III* double KO in two isogenic lines of Cambodian parasites with variable *plasmepsin* copy numbers. Disruption of *plasmepsin II* and/or *plasmepsin III* resulted in increased parasite sensitivity to PPQ, as measured by the AUC. We tested several hypotheses as to how increased *plasmepsin* copy number could influence survival under high PPQ pressure. We show that protease inhibitors have a minimal effect on parasite susceptibility to PPQ. In addition, hemoglobin digestion was not significantly increased in parasites with higher *plasmepsin* copy numbers nor did these parasites produce different amounts of free heme upon PPQ treatment. However, hemoglobin digestion was slowed in parasites with *plasmepsin II* deletions. To explore how the physiological conditions of the DV contribute to PPQ susceptibility, we treated parasites with modulators of DV function, with results suggesting that fluctuations in DV pH affect parasite response to PPQ. Thus, in Cambodian parasites, we describe the critical role of **plasmepsin* II* and **plasmepsin* III* CNVs in modulating parasite response to PPQ and begin to probe the molecular and physiological underpinnings of PPQ resistance.

## Results

### Generation of *plasmepsin* KOs in TRAC isolates

**Fig 1:**
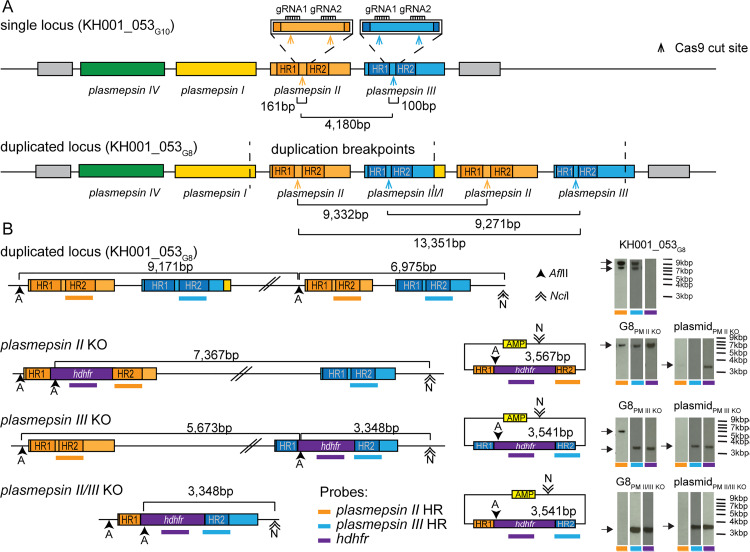
Correct disruption of the duplicated *plasmepsin* locus by KO constructs. **(A)** Schema of the single and duplicated *plasmepsin* loci predicted by Amato et al. [16]. **(B)** Schema of duplicated locus, edited loci and homology plasmids used for integration into the locus. Restriction enzyme sites and expected band sizes for Southern blots are indicated in the schema. gDNA was digested [*Afl*II (A) and *Nci*I (N)], run on a gel, transferred to a membrane, and hybridized with three different probes indicated by colored bars (orange: *plasmepsin II*, blue: *plasmepsin III,* and purple: *hdhfr* cassette). Arrows indicate expected band sizes. Southern blots for additional clones and the single locus parasites (KH001_053_G10_) are shown in S1–S3 Figs.

We have previously shown that clones from the TRAC isolate KH001_053 contain variations in *plasmepsin II* and *III* copy numbers that correlate with their PPQ resistance phenotype, as measured by the AUC in PPQ growth assays. These clones are genetically identical, including the *pfcrt* (Dd2 like) and the *pfmdr1* loci (single copy); the only difference is the *plasmepsin II/III* CNV [29]. We confirmed the tandem arrangement of the duplicated *plasmepsin II* and *III* locus [16,40], where the break points of the duplication are in the 3’ regions of *plasmepsin I* and *plasmepsin III*, resulting in a chimeric *plasmepsin III/I* between two *plasmepsin II* copies, followed by an intact *plasmepsin III* copy (Fig 1A).

To determine if either *plasmepsin II* or *plasmepsin III* is responsible for the increased AUC in PPQ growth assays, we generated *plasmepsin II* and *plasmepsin III* single KOs and a *plasmepsin II/III* double KO in the relevant genetic background of a TRAC isolate. A PPQ-susceptible clone of KH001_053 with a single copy of each *plasmepsin II* and *III* (KH001_053_G10_) and a PPQ-resistant clone with two copies of both *plasmepsin II* and *plasmepsin III* (KH001_053_G8_) served as parental lines [29]. We used CRISPR-Cas9 technology to introduce double-strand breaks in the *plasmepsin* genes and provided the parasites with a template to disrupt either *plasmepsin II* (G10_PMII_KO_ and G8_PMII_KO_) or *plasmepsin III* (G10_PMIII_KO_ and G8_PMIII_KO_) alone or both genes simultaneously (G10_PMII/III_KO_ and G8_PMII/III_KO_) with an *hdhfr* selectable marker as described previously [34]. Gene editing in the duplicated KH001_053_G8_ locus resulted in the same outcome as the KH001_053_G10_ single locus due to Cas9 cutting both copies of the duplicated *plasmepsin* genes and fusion of the two remaining pieces with the *hdhfr* marker (Figs 1B and S1–S3).

Transgenic parasites were cloned, and subsequently, the integration status was verified by PCR, quantitative PCR (qPCR) (S1 Table), and Southern blots for at least two clones per transfection (Figs 1 and S1–S3).

### Loss of *plasmepsin* duplication in field isolates abolishes PPQ bimodal response

**Fig 2:**
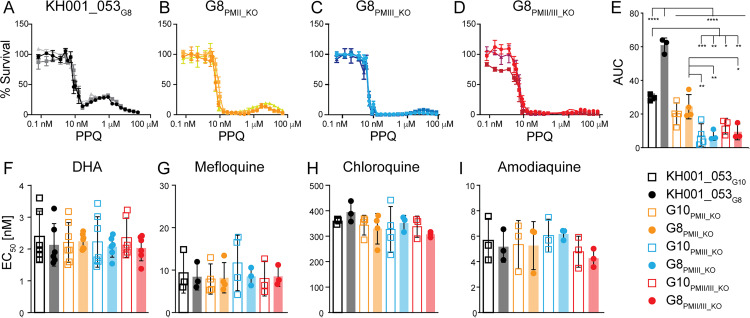
Disruption of *plasmepsin* loci leads to loss of AUC. Parasites were exposed to increasing levels of PPQ for 84 h and survival was measured by increased DNA content. Shown is an example of three biologically independent experiments run in triplicate for the KH001_053_G8_ parental locus **(A)** and parasites with disruption of either *plasmepsin II* (G8_PM II_KO_, **B)**, *plasmepsin III* (G8_PM III_KO_, **C)**, or a double KO of *plasmepsin II/III* (G8_PM II/III_KO_, **D)**. The areas under the curve (AUC) between the local minima were calculated and the average and SD are shown in for KH00_053_G10_ and KH001_053_G8_ parents as well as KOs **(E)**. Statistics show one-way ANOVA with Tukey post-test: *p < 0.05; **p < 0.01; ***p < 0.001; ****p < 0.0001. Standard EC_50_ values for the PPQ partner drug DHA **(F)** and three PPQ analogs (mefloquine **(G)**, chloroquine **(H)**, and amodiaquine **(I)**) are shown as the average and SD for at least three biological replicates run in triplicate. The two parental lines and their relative *plasmepsin* KO lines are shown. No statistically significant differences were detected between the lines.

We tested the KH001_053_G10_ and KH001_053_G8_ parental lines and the engineered *plasmepsin* KO parasites in PPQ growth assays, compared the initial Hill slope, and measured the AUC (Fig 2A–E and S2 Table). It should be noted that the KO parasites generated from KH001_053_G8_ reduced the *plasmepsin* copy numbers from two copies, and the KH001_053_G10_ KO parasites from one copy, resulting in genetically identical KOs. Consequently, the engineered parasites from KH001_053_G10_ and KH001_053_G8_ were also indistinguishable in their phenotypic response (Fig 2E, one-way ANOVA with Tukey post-test *p* < 0.05). We therefore used the KH001_053_G8_ KO lines for further phenotypic analysis. G8_PMII_KO_, G8_PMIII_KO_, and G8_PMII/III_KO_ had indistinguishable initial Hill slopes but had a strong significant reduction of the AUC when compared to the KH001_053_G8_ parent with two copies of both *plasmepsin II* and *III* (Fig 2A–D, one-way ANOVA with Tukey post-test *p* < 0.0001). The same was true for G10_PMII_KO_, G10_PMIII_KO_, and G10_PMII/III_KO_ compared to KH001_053_G8_. However, only G8_PMIII_KO_, G8_PMII/III_KO_, G10_PMIII_KO_, and G10_PMII/III_KO_ showed significantly reduced AUC compared to the KH001_053_G10_ single locus (Fig 2E and S2 Table, one-way ANOVA with Tukey post-test *p* < 0.05). Taken together, these data confirm the role of duplicated *plasmepsin* genes in survival of parasites at high concentrations of PPQ and that expression of *plasmepsin III* appears to be the dominant driver of this phenotype.

### Loss of plasmepsins does not affect susceptibility to other antimalarials

To understand if *plasmepsin* CNVs modulate susceptibility to other antimalarials, we also subjected the engineered *plasmepsin* KO lines to a panel of related quinolines and DHA (*i.e.*, the partner drug of PPQ in ACTs) in standard *in vitro* drug susceptibility assays. There were no significant differences in EC_50_ between the parental lines and the clonal *plasmepsin* KO lines for chloroquine (CQ), mefloquine (MEF), amodiaquine (AQ) or DHA (Fig 2F–I and S2 Table; one-way ANOVA with Tukey post-test, *p* > 0.05). We used EC_50_ assays rather than ring-stage survival assays (RSA) to test DHA susceptibility as we did not see a correlation between *plasmepsin II* and *III* copy numbers and RSA phenotype in parasites with the same genetic background as the parental lines we used in previous studies [29]. As expected for parasites with a Dd2-like *pfcrt*, the parental lines and *plasmepsin* KO lines were all resistant to CQ compared to the CQ-sensitive laboratory strain 3D7 (EC_50_: 12 nM, S2 Table). These data suggest that the role of *plasmepsin* copy numbers in increased survival is specific to PPQ and does not extend to other closely related quinolines.

### Aspartic and cysteine protease inhibitors do not impact the PPQ AUC

Given that deletion of *plasmepsin II* and *plasmepsin III* abolished the PPQ-resistance phenotype, we wondered if direct inhibition of the protease function of Plasmepsin II and III might reduce the AUC. We demonstrated that the protease inhibitors E64 (a broad-band cysteine protease inhibitor shown to inhibit Falcipain-2 in the DV [41] and block parasite egress from the host red blood cell [42]) and pepstatin A (pepA) (an aspartic protease inhibitor shown to inhibit Plasmepsin function in cell lysates and to bind to Plasmepsin II [43]) had no differential activity in parasites regardless of *plasmepsin* copy number (S2 Table).

We next investigated if there was an additive effect of the protease inhibitors in combination with different PPQ concentrations in parasites with increased *plasmepsin* copy numbers. We identified two different drug concentrations where parasite growth was affected but not severely inhibited for each protease inhibitor (69–89% growth, S4 Fig). We then exposed parasites to a constant concentration of each protease inhibitor (7.5 μM and 5 μM for pepA; 2.6 μM and 3.9 μM for E64) in the presence of increasing concentrations of PPQ. Measurements of parasite growth in these assays were normalized to parasite growth with only the protease inhibitor present. We tested the parental lines with the single and the duplicated locus as well as an additional TRAC isolate with an even higher AUC (KH004_057, AUC = 88 ± 21 [29]). The tested protease inhibitor concentrations had no significant effect on the AUC (*p* > 0.05, one-way ANOVA followed by Tukey post-test), suggesting that disruption of hemoglobin catabolism by protease inhibitors does not modulate PPQ susceptibility under the conditions tested here (S5 Fig, Table A in S3 Table).

### PPQ-induced heme accumulation is not impacted by *plasmepsin II or III* copy numbers

To further explore the possible mechanism of PPQ resistance, we tested the effect of PPQ on hemozoin biocrystallization using a pyridine-labeled heme fractionation assay [44,45]. This assay uses a series of cellular fractionation steps to extract the different heme species in the parasites (*i.e.*, hemoglobin, free heme, and hemozoin) and subsequently measures the Fe^3+^-heme-pyridine absorbance. It has been demonstrated previously that parasites exposed to increasing CQ or PPQ concentrations show increased levels of free heme (and to a lesser degree hemoglobin) and reduced levels of hemozoin [21].

**Fig 3:**
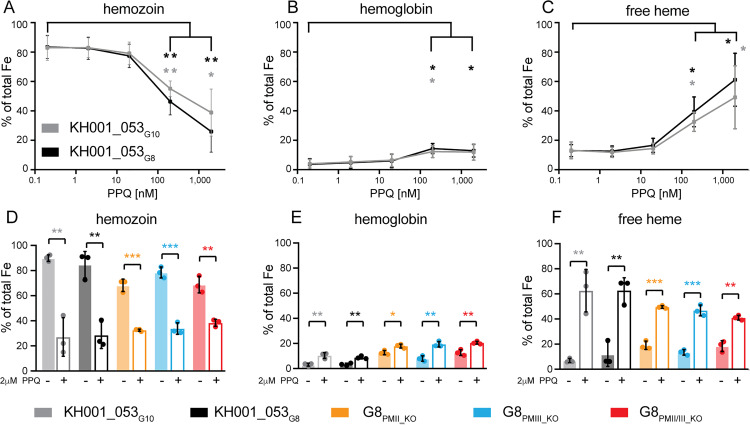
Heme fractionation of PPQ-treated and untreated parasites with variable *plasmepsin* copy numbers. Different heme species were extracted from parasites by subsequent cellular fractionation steps. **(A-C)**: Tightly synchronized KH001_053_G10_ (grey) and KH001_053_G8_ (black) ring stage parasites were exposed to various PPQ concentrations for 32 h. Average and SD of percentage of hemozoin Fe **(A)**, hemoglobin **(B)**, and free heme Fe **(C)** are shown for three independent experiments run in quadruplicate. Statistical comparisons of the drug-treated lines to their untreated controls were performed using two-tailed unpaired Student’s t-tests *p < 0.05; **p < 0.01. **(D-F)**: KH001_053_G10_ and KH001_053_G8_ parental lines as well as G8_PMII_KO_, G8_PMIII_KO_, and G8_PMII/III_KO_ were incubated with 2 μM PPQ from 24 to 36 h post-synchronization and harvested at 36 h. Statistical comparisons of treated to untreated parasites at the 36 h timepoint from three independent biological replicates were performed using two-tailed unpaired Student’s t-tests *p < 0.05; **p < 0.01; ***p < 0.001.

To understand if the additional copies of *plasmepsin II* and *III* influence the generation of free heme or the degradation of hemoglobin under PPQ pressure, we exposed highly synchronized ring-stage parasites (0–6 h post-invasion) from the KH001_053_G8_ and KH001_053_G10_ parental lines to a range of PPQ concentrations for 32 h and fractionated the different heme species. We then determined the percentage of each heme species present in the total amount of iron extracted. On average, both parental lines showed similar percentages of all heme species in the absence of drug (average of 13% free heme, 4% hemoglobin, and 83% hemozoin, Fig 3A–C and S4 Table). As shown previously [21], parasites exposed to 200 nM or 2 μM PPQ showed a significant increase in free heme (and to a lesser extent hemoglobin) compared to the untreated control, as well as a reduction in hemozoin formation (unpaired Student’s t-test *p* < 0.05). These findings are consistent with an inhibition of heme detoxification and hemozoin formation by PPQ treatment. There was no significant difference in the proportion of any heme species between the parental lines containing one (KH001_053_G10_) or two (KH001_053_G10_) copies of *plasmepsin II* and *III*. This suggests that while both parasite lines experience similar levels of toxic free heme, parasites with increased *plasmepsin* copy numbers seem to be better adapted to survive these extremely high concentrations of PPQ or free heme.

We next exposed parental and KH001_053_G8_ KO parasites (G8_PMII_KO_, G8_PMIII_KO_, and G8_PMII/III_KO_) to a single dose of 2 μM PPQ for 12 h between 24 and 36 h post-invasion when hemoglobin digestion is highest. As seen before, even a 12 h exposure of parasites to 2 μM PPQ led to a significant decrease in hemozoin, and an increase in free heme and hemoglobin compared to the 36–42 h untreated control in all lines tested (Fig 3D–F and S5 Table, unpaired Student’s t-test, *p* < 0.05). However, free heme accumulation was higher in the parental lines (>60%) than in the KO lines (<50%), suggesting that less Plasmepsin II and III activity leads to less free heme. This demonstrates that PPQ is inhibiting hemozoin formation regardless of Plasmepsin II and III abundance or presence.

### Hemoglobin-derived peptides are not altered in parasites with increased genomic copies of *plasmepsin II* and *III*

While the heme fractionation assay can determine the relative abundance of different heme-iron species, it is not quantitative. We examined whether the abundance of hemoglobin-derived peptides differed between the lines, as previously shown for parasites carrying *Pf*CRT mutations that confer PPQ resistance [22]. The *P. falciparum* RF7 clinical isolate has three copies of each *plasmepsin II* and *III* and a *pfcrt* M343L mutation; this mutant residue is located on the cytosolic side of *Pf*CRT and is associated with decreased PPQ sensitivity and altered peptide abundance as compared to Dd2-like *Pf*CRT [22]. To explore whether *plasmepsin* CNV could influence peptide composition, we subcloned RF7, generating two clonal lines, one with a single copy of *plasmepsin II* and *III* (B9) and a second clonal line with three copies of *plasmepsin II* and *III* (D4). The *Pf*CRT M343L mutation is present in each clone. We performed peptidomic analysis on these clones and ran the analysis in both positive and negative mode. We detected a total of 35 putative endogenous hemoglobin-derived peptides (dipeptides to 13-mers) that could be mapped to either the α or β chains of hemoglobin (S6 Table). We did not observe any significant difference in hemoglobin-derived peptide composition or abundance when comparing parasite clones with single (B9) or triple (D4) *plasmepsin II* and *III* copy numbers (S6A and S6B Fig). These data further suggest that hemoglobin digestion is not significantly altered in the presence of increased *plasmepsin* copy numbers.

To better understand what effects PPQ treatment has on parasite metabolism, we used targeted metabolite analysis of 3D7 trophozoites treated for 2.5 h with 140 nM PPQ or 10 nM ATQ (as a control, S7 Table and S6C and S6D Fig). Parasites treated with ATQ, a known inhibitor of the electron transport chain, showed a strong increase in N-carbamoyl-L-aspartate and dihydroorotate as expected (S6D Fig) [46]. In contrast, there were no significant changes in our targeted list of metabolites in parasites treated with PPQ compared to untreated parasites (S6C Fig). Similarly, untargeted analysis of all putative hemoglobin-derived peptides (≤13 amino acids in length) clearly shows that there is little difference in the putative peptide signals between PPQ-treated and mock-treated parasites (S6E and S6F Fig and S8 Table).

### Hemoglobin digestion is slowed in *plasmepsin* KO parasites

**Fig 4:**
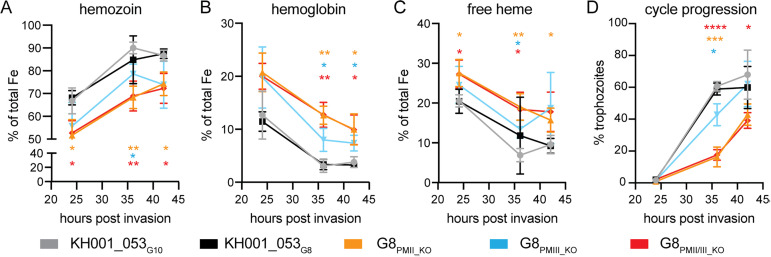
*Plasmepsin* KO parasites have slowed hemoglobin metabolism. Tightly synchronized parasites were harvested at different time-points throughout the life cycle. The average and SD of percentage of hemozoin Fe **(A)**, hemoglobin **(B)**, and free heme Fe **(C)** are shown for three independent experiments for parasites with a single *plasmepsin* locus (KH001_053_G10,_ grey), duplicated *plasmepsin* locus (KH001_053_G8,_ black), G8_PMII_KO_ (orange), G8_PMIII_KO_ (blue), or G8_PMII/III_KO_ (red). Statistical comparisons at each time point were performed between the single copy KH001_053_G10_ line and all other lines using two-tailed unpaired Student’s t-tests *p < 0.05; **p < 0.01. **(D)** Cell cycle progression was measured by the number of nuclei present per cell using flow cytometry and SYBRGreen staining of DNA. Parasites were defined as trophozoites when they had at least three nuclei and had started DNA replication. Shown is the percentage of trophozoites at each time point (24 h, 36 h and 42 h post-invasion). Statistical comparisons were performed between the single copy KH001_053_G10_ line and all other lines at the same time point using two-tailed unpaired Student’s t-tests *p < 0.05; ***p < 0.001; ****p < 0.0001.

To further explore the role of Plasmepsins in hemoglobin degeneration, we investigated whether parasites with increased copy numbers or deletions of *plasmepsin II* and *III* differ in their composition or accumulation of heme species during their life cycle. We tightly synchronized the two parental lines KH001_053_G10_ and KH001_053_G8_ as well the KO clones G8_PMII_KO_, G8_PMIII_KO_, and G8_PMII/III_KO_ and performed heme fractionation assays at timepoints 24, 36, and 42 h post-synchronization (Fig 4). As expected, the hemozoin percentage increased over time for all lines, while hemoglobin and free heme concentrations decreased (Fig 4A–C and S5 Table). Throughout the lifecycle, there was a significantly lower proportion of hemozoin in the G8_PMII_KO_ and G8_PMII/III_KO_ lines compared to the KH001_053_G10_ single copy line (Fig 4A, unpaired Student’s t-test *p* < 0.05). Concordantly, hemoglobin and free heme levels were significantly higher in the G8_PMII_KO_ and G8_PMII/III_KO_ lines compared to KH001_053_G10_, suggesting that hemoglobin digestion is slowed in the absence of *plasmepsin II.* G8_PMIII_KO_ parasites showed an intermediate phenotype between the single copy parent KH001_053_G10_ line and the G8_PMII_KO_ and G8_PMII/III_KO_ lines (Fig 4A-C).

A similar increase in undigested hemoglobin has been observed in PPQ-resistant parasite lines carrying the *Pf*CRT G353V or F145I variants [22] as well as in *plasmepsin II* KOs examined by Western blot [36]. There was no difference between the two parental lines, suggesting that hemoglobin digestion efficiency is not increased in parasites with additional *plasmepsin* copy numbers. There was no statistically significant difference in individual heme species of a parasite line between the time point at 36 and 42 h (by unpaired Student’s t-test), indicating that most of the hemoglobin digestion was completed by 36–42 h post-invasion. The reduced hemoglobin digestion efficiency in KO parasites could explain the observation that less free heme is released under PPQ treatment (Fig 3F).

We next asked whether the delay in hemozoin formation seen in G8_PMII_KO_ and G8_PMII/III_KO_ was correlated to a delay in overall life cycle progression. To measure cell cycle progression, we determined the percentage of trophozoites in each sample by flow cytometry prior to the heme-iron species extraction (Fig 4D). In agreement with the heme-iron species data, trophozoite formation was delayed in the *plasmepsin* KO parasites compared to both KH001_053_G10_ and KH001_053_G8_ parental lines, with the G8_PMII_KO_ and G8_PMII/III_KO_ lines being more delayed than the G8_PMIII_KO_ line. It remains unclear whether the delay in hemoglobin digestion is the result or the cause of slowed progression through the life cycle.

### Growth in amino acid-limited media does not change PPQ sensitivity

**Fig 5:**
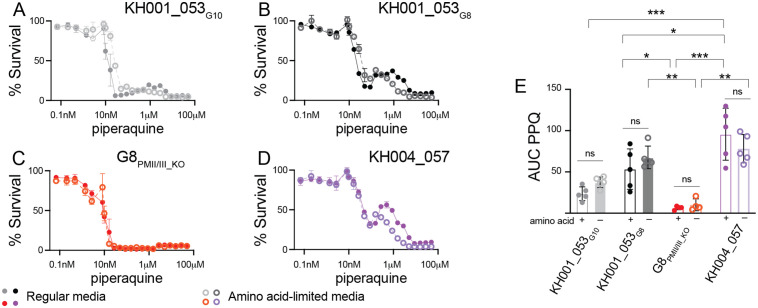
Low amino acid conditions do not markedly impact the PPQ resistance phenotype. Parasites were exposed to increasing levels of PPQ in regular (dots and solid lines) or amino acid-limited media (circles and dotted lines) for 84 h and survival was measured by increased DNA content. Shown is an example of at least four biologically independent experiments run in triplicate for the single copy *plasmepsin* clone KH001_053_G10_ **(A)**, the duplicated *plasmepsin* locus clone KH001_053_G8_ **(B)**, the double KO of *plasmepsin II/III* (G8_PM__II/III_KO_, **C)**, and the multicopy *plasmepsin* locus clone KH004_057 **(D)**. The area under the curve (AUC) between the local minima was calculated and the average and SD is shown in **(E)**. Statistics show two-way ANOVA with Šidák correction: *p < 0.05; **p < 0.01; ***p < 0.001; ns: not significant.

We compared PPQ sensitivity between parasites cultured in regular media and those cultured in amino acid-limited media. We used a mixture of 25% complete RPMI and 75% RPMI free of all amino acids except isoleucine, methionine, and glutamine as amino acid-limited media (S7 Fig). We then performed PPQ drug inhibition assays with the KH001_053_G10_, KH001_053_G8_, G8_PMII/III_KO_ and KH004_057 lines in regular and amino acid-limited media. All four lines showed similar PPQ phenotypes in amino acid-limited media compared to regular media. The KH001_053_G8_ and KH004_057 parasite lines with increased *plasmepsin II* and *III* copies had significantly higher AUC than the *plasmepsin* double KO parasite G8_PMII/III_KO_ in both media conditions (Table B in S3 Table and Fig 5, two-way ANOVA with Šidák correction). The size of the AUC therefore still correlates with *plasmepsin II* and *III* copy number under amino acid-limited conditions.

We also tested replication levels of KH001_053_G10_, KH001_053_G8_, G8_PMII/III_KO,_ and KH004_057 in regular media and amino acid-limited media, and all four lines showed similar replication patterns to each other (S8 Fig). In regular media, parasitemia increased 6-to-7-fold with no statistical difference between the lines (one-way ANOVA with Tukey post-test). In contrast, all parasite lines grew significantly less in amino acid-limited media indicated by an increase in parasitemia of 2-fold only (S8 Fig). These replication rates suggest that all lines still rely similarly on amino acid uptake from the media regardless of *plasmepsin* copy number.

### PPQ activity is not dependent on external pH

Drug uptake and availability to the parasite can be affected by the drug’s protonation status and its ability to cross membranes. Changes in the pH of the extracellular environment have been shown to affect CQ potency [47,48]. CQ is membrane permeable at neutral pH, but loses its permeability once protonated in the low pH of the DV [49] and concentrates within the DV via ‘weak-base trapping’ and subsequent binding to ferriprotoporphyrin IX [50]. Decreasing the pH of the extracellular environment reduces the overall pH gradient between the DV and extracellular environment and leads to reduced CQ accumulation in the DV and increased survival of the parasites.

Indeed, in media with a pH adjusted to 6.74, EC_50_ values for CQ are dramatically increased when compared to the EC_50_ values at a neutral pH of 7.5 (S9A–F Fig and S9 Table). Moreover, Dd2, KH001_53_G8_, and KH001_53_G10_ were not completely killed at the highest CQ concentration tested (2 μM). Increasing the pH of the media to 8.24 had the opposite effect, significantly reducing the EC_50_ of CQ when compared to media at a pH of 7.5 (*p* > 0.01, paired Student’s t-test, S9 Fig and S9 Table). This is consistent with a larger pH gradient between the extracellular medium (at pH 8.24) and the DV leading to increased CQ accumulation within the DV.

**Fig 6:**
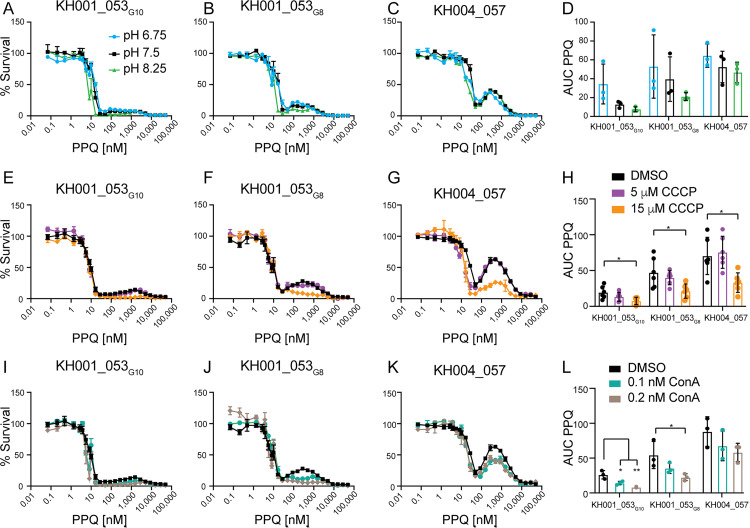
Effect of pH on PPQ AUC. **A-D:** Parasites were exposed to increasing levels of PPQ for 84 h in acidic (pH = 6.74), normal (pH = 7.5) or basic (pH = 8.24) media. Shown is an example of three biologically independent experiments run in triplicate for KH001_053_G10_ **(A)**, KH001_053_G8_ **(B)** or KH004_057 **(C)**, as well as the average and SD of the AUC for all three biological replicates **(D)**. There were no statistically significant differences detected. **(E-L):** Parasites were exposed to increasing levels of PPQ in the presence of DMSO or **E-H)** CCCP at either 5 μM or 15 μM concentrations or **I-L)** concanamycin A (ConA) at either 0.1 nM or 0.2 nM. Shown is one example of three biologically independent experiments run in triplicate for KH001_53_G10_ **(E,** **I)**, KH001_53_G8_ **(F, J)**, and KH004_057 **(G,** **K)**. The area under the curve (AUC) between the local minima was calculated and the average and SD are shown for CCCP **(H)** and ConA **(L)**. Statistical comparisons of the drug-treated lines and DMSO-treated controls were performed using one-way ANOVA with Dunnett’s post-test: *p < 0.05 and **p < 0.01.

PPQ, like CQ, is a weak base, and we hypothesized that its protonation could be affected by the pH of its surrounding medium. In *in vitro* PPQ susceptibility assays, parasites showed only marginal higher sensitivity to high PPQ concentrations at increased extracellular pH, and the differences in AUC between the different pH conditions were not statistically significant (Fig 6A–D and S9 Table). This suggests that the activity of PPQ is less affected by external pH than the CQ activity.

### Alkalization of the DV affects PPQ AUC

The hemoglobin digestion activity of DV lysates [32,51], as well as recombinant Plasmepsins [30,31], is maximal at low pH, consistent with conditions within the acidic DV (DV pH of about 4.5–5.5 [52–54]). We therefore reasoned that perturbations to the DV pH might also impact Plasmepsin activity and thereby alter the PPQ resistance phenotype of parasites with increased *plasmepsin II and III* copy numbers. We used concanamycin A (ConA, an inhibitor that blocks the V-type ATPase from pumping H^+^ ions into the DV lumen, thereby leading to an alkalization of the DV [55]) and carbonyl cyanide m-chlorophenyl hydrazone (CCCP, a proton ionophore dissipating H^+^ gradients across membranes in general) to interrogate the role of the DV pH in PPQ resistance.

We first determined the EC_50_ for ConA and CCCP. While we did not detect any differences in susceptibility to ConA, G8_PMII_KO_ and G8_PMII/III_KO_ parasites were less susceptible to CCCP than KH001_053_G10_ (*p* < 0.05, one-way ANOVA followed by Dunnett’s post-test, Table C in S3 Table). Previous work demonstrated that vacuolar pH impacted CQ efficacy [48,56], and we similarly found a significant increase in susceptibility to CQ when we tested CQ in the presence of CCCP at a concentration of either 5 μM or 15 μM against parasites lines with various *plasmepsin* copy numbers (*p* < 0.01, one-way ANOVA followed by Tukey post-test, S9G-L Fig and Table D in S3 Table). Interestingly, the decreased survival was specific for the CQ-resistant *Pf*CRT isoform and had non-significant effect on the CQ-sensitive 3D7 parasite line.

Similarly, when treated with 15 μM CCCP and normalized to growth in 15 μM CCCP without PPQ, parasites tested in an PPQ susceptibility assay exhibited reduced AUC values compared to parasites not treated with CCCP. This finding indicates that intracellular pH also affects the PPQ resistance phenotype (*p* < 0.05 one-way ANOVA followed by Dunnett’s post-test, Fig 6E–H and Table A in S3 Table). When we tested ConA in combination with PPQ, we also found a significant reduction in the AUC for KH001_053_G10_ and KH001_053_G8_ but not for KH004_057 (Fig 6I–L and Table A in S3 Table). We next wanted to test whether PPQ itself could affect the alkalization of the DV.

### PPQ does not influence DV pH

**Fig 7:**
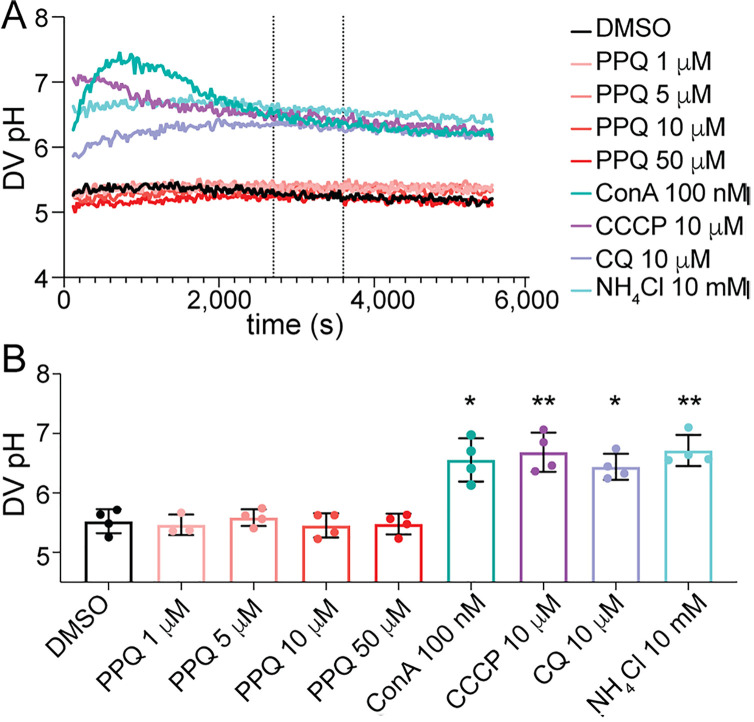
Digestive vacuole pH remains unchanged with exposure to various concentrations of PPQ. **(A)** DV pH traces of Dd2 parasites exposed to PPQ and control treatments of concanamycin A (ConA, 100 nM), CCCP (10 µM), CQ (10 µM) and NH_4_Cl (10 mM). Shown are the averaged results of internal technical duplicates from a representative experiment. DV pH was quantified for each treatment as an average of the measurements taken between 45 and 60 mins of compound exposure (dashed vertical lines), **(B)** average DV pH measurements following 45 to 60 mins of exposure to PPQ at 1, 5, 10 or 50 µM and to known DV pH modulators ConA, CQ, CCCP and NH_4_Cl. The data are the average and SD of three to four independent experiments (performed with blood from different donors). The asterisks denote a significant difference from the DMSO solvent control: *p <* *0.05; **p <* *0.01, (one-way ANOVA). Raw data are provided in S10 Table.

The weak-base trapping effect of CQ can cause alkalinization of acidic compartments [57]. The activities of Plasmepsin enzymes are pH-sensitive and are most active at low pH. Hence, we hypothesized that if PPQ also acts as a weak base accumulator, sufficiently high concentrations of PPQ in the DV might disrupt the DV pH, thereby reducing Plasmepsin activity, and the production of free heme that leads to parasite death. Amplification of *plasmepsin II* and *III* could therefore rescue this reduced activity due to increased protein expression, leading to the survival of PPQ-resistant parasites under high PPQ concentrations. To test this hypothesis, we used ratiometric fluorescence-based measurements of saponin-isolated Dd2 trophozoite parasites with fluorescein-dextran loaded DVs as a way to monitor DV pH in the presence or absence of PPQ. Fluorescence traces for pH calibration buffers indicated resting DV pH values of 5.5 ± 0.2 (mean ± SD, n = 4; Fig 7), consistent with previous studies [53,58]. The V-type ATPase inhibitor ConA (100 nM), proton ionophore CCCP (10 µM), and weak-base NH_4_Cl (10 mM) all caused rapid alkalinization of parasite DVs, resulting in significantly elevated DV pH compared to the vehicle control following 45 min of exposure (6.5–6.7; *p* < 0.05, one-way ANOVA with Dunnett’s post-test, Fig 7 and S10 Table). CQ at 10 µM also caused significant alkalinization, resulting in an average DV pH of 6.4 ± 0.2 (*p* < 0.05, one-way ANOVA, Dunnett’s post-test). By contrast, concentrations of up to 50 µM of PPQ had no impact on DV pH (Fig 7), suggesting that PPQ does not exert an effect via pH modulation over this time-course. This provides further evidence that PPQ and CQ have distinct effects on DV physiology.

## Discussion

We explored the contribution of *plasmepsin* CNVs to the bimodal resistance phenotype to PPQ as measured by the AUC in *P. falciparum* parasites, independent of the known contributions of *pfcrt* mutations. We generated *plasmepsin II, III,* and double KO parasites in the clinically relevant genetic background of Cambodian isolates. Consistent with previous findings in different genetic backgrounds, Plasmepsins are not essential for parasite survival under *in vitro* conditions [34,37,59,60]. In PPQ dose-response experiments however, the resulting *plasmepsin* KO parasite lines had lost their secondary survival peak, clearly demonstrating that Plasmepsins are a main driver of the AUC phenotype.

KO lines demonstrated a slower rate of hemoglobin degradation and delayed cell cycle progression compared to wildtype parasites but were similarly susceptible to the protease inhibitors E64 and pepA, consistent with prior studies [30–34,36]. While other *Plasmodium* species have only one aspartic protease in the DV, *P. falciparum* has expanded the repertoire from the homolog *plasmepsin IV* to three additional paralogs including *plasmepsin II* and *III* [61]. Earlier studies showed that there is no significant change in expression of the other Plasmepsins or falcipains in individual *plasmepsin* KO lines [34]. It is likely that the paralogous Plasmepsins can perform similar or overlapping roles; as such, we expect that Plasmepsin I and/or IV can partially compensate for Plasmepsin II and/or III loss.

We investigated several potential biochemical and metabolic mechanisms underlying the AUC phenotype. However, we were unable to define a specific mechanism explaining the genetic results. Multiple copies of *plasmepsin II* and *III* did not increase hemozoin formation and parasites with *plasmepsin* CNVs showed no difference in levels and composition of hemoglobin-derived peptides. Furthermore, the addition of PPQ showed similarly high levels of free heme and reduced hemozoin formation in single and multicopy *plasmepsin* parasites. This lack of impact of *plasmepsin II and III* copy numbers on hemoglobin digestion was also evident when parasites were grown under amino acid-constrained conditions, which places greater reliance on hemoglobin degradation for amino acid metabolism. There was little difference in growth between parasite lines grown in amino acid-limited media, and susceptibility to PPQ was not impacted by amino acid-restriction in parasites with increased copy numbers or *plasmepsin* KO. Together, these observations suggest that hemoglobin degradation is tightly regulated by the parasite, and simple upregulation of this pathway by *plasmepsin II* and *III* amplification cannot explain the PPQ resistance mechanism.

We also hypothesized that PPQ accumulation might impact DV pH and thereby modulate the activity of the aspartic proteases in the DV. In accordance with this hypothesis, survival under PPQ pressure was slightly reduced when the DV pH was disturbed by proton ionophores or V-type ATPase inhibitors. However, in contrast to the pH buffering effect observed by us and others for CQ [57], PPQ had no effect on DV pH at a range of physiological concentrations. These data suggest that PPQ does not exert a pH buffering effect on the DV. In contrast, we observed that CQ sensitivity is highly responsive to perturbations of the parasite proton gradient by altered extracellular pH or the addition of proton ionophores or V-type ATPase inhibitors, consistent with previous studies [48,50,62]. CQ and PPQ are weakly basic and are expected to accumulate to high concentrations in the DV, however, PPQ is substantially more lipophilic than CQ (logP of 6.1 for PPQ and 4.7 for CQ), and has a lower mean pKa value than CQ (6.1 vs 9.3) [63,64]. Together, this leads to an 8 orders-of-magnitude greater predicted lipid accumulation ratio for PPQ (~973,000) compared to CQ (8.25) under the acidic conditions of the DV [63,64]. Thus, it is possible that high concentrations of PPQ are sequestered within membranes and lipids of the DV, perhaps including the lipid droplets within which hemozoin formation is thought to occur [65,66]. This would be consistent with the lack of a direct effect of PPQ on DV pH, in contrast to CQ.

The unusual biphasic curve exemplified by PPQ-resistant parasites suggests that multiple competing processes may be underlying this complex phenotype. Indeed, *plasmepsin* copy number amplification is linked to two other determinants of drug resistance, mutations in *pfcrt*and *kelch13*. The Cambodian isolates studied here possess the Dd2 isoform of *Pf*CRT (which provides CQ resistance but not PPQ resistance) and carry the Kelch13 C580Y allele that confers delayed clearance by DHA. Both genes are implicated in DV-related processes. Kelch13 has been localized to the cytostome where it plays a role in hemoglobin endocytosis and trafficking to the DV, and the C580Y mutation present in the Cambodian isolates studied here has been shown to reduce hemoglobin uptake in early-stage parasites [67,68]. It is possible that the increase in *plasmepsin* copy numbers may have arisen in this background due to DHA-PPQ treatment pressure in Southeast Asia and could play a compensatory role by increasing hemoglobin degradation in early rings. Indeed, the inheritance of Kelch13 C580Y has been linked with increased *plasmepsin* copy numbers in a genetic cross between a wildtype Malawian parasite and a Cambodian line [69]. Furthermore, increased *plasmepsin* copy numbers have been observed in field isolates in Southeast Asia in a large diversity of genetic backgrounds, independent of DHA-PPQ selection pressure [27], as early as 2007 [25,70]. Unfortunately, we were unable to reliably measure hemozoin formation in early ring stages and therefore could not directly test whether *plasmepsin* amplification compensates for delayed hemoglobin degradation in *kelch13* mutants.

*Pf*CRT is a DV-resident transporter required for the transport of hemoglobin-derived peptides out of the DV [22,71–73]. Mutations in *Pf*CRT can mediate drug efflux from the DV [22,74–77]. The CQ-resistant, PPQ-sensitive Dd2 haplotype of *pfcrt* is present in all parasites in this study and carries 8 amino acid differences from the canonical 3D7 wildtype allele (*i.e.*, M74I, N75E, K76T, A220S, Q271E, N326S, I356T, and R371I). No additional *pfcrt* mutations exist in these parasites, so differences in PPQ resistance between *plasmepsin* KO, *plasmepsin* single-copy, and *plasmepsin* multicopy parasites are attributed to *plasmepsin* CNVs and not to *pfcrt* mutations. We hypothesize that *plasmepsin* amplifications need a mutated Dd2-like *pfcrt* background to confer PPQ resistance, as *plasmepsin* amplification in wildtype *pfcrt* parasites does not lead to PPQ resistance [78]. Novel mutations added to the Dd2-isoform of *Pf*CRT (e.g., C101F, T93S, H97Y, F145I, I218F, M343L, and G353V) can also mediate PPQ resistance in the absence of *plasmepsin* copy number changes in natural isolates [2,5,19,25] and engineered parasites [20,21,28,79], and can be additive or synergistic with *plasmepsin* CNVs. Two genetic crosses with a sensitive parasite and a PPQ-resistant parasite harboring increased *plasmepsin* copy number and a novel *pfcrt* mutation have been generated and analyzed for their PPQ phenotypes [69,80]. While the major contributor to high PSA survival was mapped to *pfcrt*, higher copy numbers of *plasmepsin II* and *III* increased the resistance even further. When AUC was used as a phenotype for QTL mapping, the main peak was *pfcrt* but a second strong peak was found around *plasmepsins*, suggesting an epistatic link between *Pf*CRT and Plasmepsins in conferring PPQ resistance [80].

Heterologous expression studies show that Dd2 *Pf*CRT is capable of transporting PPQ, albeit poorly, and PPQ resistance-conferring mutations in *Pf*CRT increase this transport activity [77,81]. Furthermore, PPQ-resistant *pfcrt* mutations confer substantial fitness costs that are associated with accumulation of hemoglobin-derived peptides [22,23,82]. It remains possible that *plasmepsin* amplification could modulate interactions between Dd2 isoforms of *Pf*CRT and hemoglobin-derived peptide substrates or between *Pf*CRT and PPQ (*e.g.,* a competitive substrate of *Pf*CRT) [77], leading to increased fitness and/or PPQ resistance. Regardless of the underlying mechanism, there is strong genetic evidence for an epistatic interaction between novel *pfcrt* mutations and *plasmepsin* CNVs in PPQ-resistant parasites.

In Southeast Asia – where increased copy numbers of *plasmepsins* have been described as early as 2007, alongside *kelch13* mutations – mutations in *pfcrt* appear to have emerged after 2010 [83] and to have risen toward fixation by 2016 in an increased *plasmepsin* copy number background [20]. However, the opposite observation was made in South America, where the *Pf*CRT^C350R^ variant has been detected since 2002, while *plasmepsin* amplifications were only detected after 2007 [24]. A follow-up study found no significant association between the *Pf*CRT^C350R^ variant and *plasmepsin* copy numbers and observed a decrease in the prevalence of both between 2016–2021 [84]. In Africa, where the proportion of *pfcrt* CQ resistant haplotypes is not fixed, increased *plasmepsin II* copy numbers have been detected in parasites collected between 2014–2015, notably in Burkina Faso and Uganda (>30%) [85]. It remains to be seen whether *plasmepsin* CNV confers some degree of resistance to PPQ in these African backgrounds, in the absence of *pfcrt* or *kelch13* mutations.

The precise mechanism for *plasmepsin* amplification-mediated PPQ resistance remains unclear. One possibility is that high levels of unbound heme or heme-drug conjugates induced by high PPQ concentrations might cause oxidative stress and impair hemoglobin degradation, such that *plasmepsin II* and *III* amplification in PPQ-resistant parasites could restore sufficient hemoglobin degrading activity for continued parasite growth in the presence of PPQ. Indeed, it has been suggested that Plasmepsin II and III exist in a hemozoin-detoxifying complex that is inhibited by CQ at both the hemoglobin degradation and hemozoin formation steps [86]. *Plasmepsin II and III* amplification could therefore influence the rate-limiting steps of this process, leading to the complex concentration-response curve observed for PPQ-resistant parasites. Furthermore, high concentrations of PPQ could potentially interfere with the transport of hemoglobin-derived peptides via *Pf*CRT. Thus, in addition to ensuring continued hemoglobin degradation under high free heme, *plasmepsin II* and *III* amplification might also ensure sufficient flux of hemoglobin-derived peptides to outcompete PPQ for *Pf*CRT-mediated transport, further ensuring continued supply of amino acids to the parasite cytosol under high PPQ exposure.

Overall, in the natural setting, *plasmepsin* CNV has a role in conferring PPQ resistance. Here, we demonstrate that the AUC phenotype (*i.e.*, increased parasite survival under high PPQ pressure) is mediated by increased *plasmepsin* copy number in TRAC isolates. While we document the importance of *plasmepsins* in mediating increased AUC in PPQ-resistant parasites, further studies are required to arrive at a complete mechanistic understanding of the biological role of *plasmepsins* in PPQ resistance.

## Materials and methods

### Ethics statement

The *Plasmodium falciparum* isolates used in this study were derived from samples collected as part of the Tracking Resistance to Artemisinin Collaboration (TRAC), which was approved by local Institutional Review Boards (IRBs) and the Oxford Tropical Research Ethics Committee (OxTREC). Written informed consent was obtained from all participants or their guardians as part of the original TRAC studies.

All *P. falciparum* cultures were maintained in human erythrocytes and serum obtained from healthy anonymous donors, purchased through Interstate Blood Bank (Memphis, TN, USA), a commercial vendor licensed to provide de-identified human biological materials for research use. No individuals were recruited or sampled as part of this study.

All laboratory procedures involving genetically modified *P. falciparum* parasites were conducted under approved Biosafety Level 2 (BSL-2) conditions in accordance with the guidelines of the Institutional Biosafety Committee (IBC) at the Harvard T.H. Chan School of Public Health.

### Parasite culture

The parental parasite lines (KH001_053 and KH004_057) were collected from Pursat and Pailin, Cambodia in 2011 through the Tracking Resistance to Artemisinin Collaboration (TRAC), and culture adapted and subcloned as previously described resulting in KH001_053_G10_ and KH001_053_G8_ [29]. All parasites were grown in fresh human erythrocytes (O+) at 4–5% hematocrit in Roswell Park Memorial Institute (RPMI) 1640 media supplemented with 26.6 mM NaHCO_3_, 27.7 mM HEPES, 0.41 mM hypoxanthine, 10% O+ human serum, and 25 μg/mL gentamicin. Human serum (heat inactivated and pooled) and human erythrocytes were supplied by Interstate Blood Bank, Inc., Memphis, TN. For *in vitro* drug susceptibility assays, parasites were cultured in media containing 0.5% Albumax (Invitrogen, Carlsbad, CA), in place of human serum. Cultures were incubated at 37°C with rotation (55 RPM) under 1% O_2_/5% CO_2_/balance N_2_ gas. For amino acid-limited conditions, Albumax media was generated as described with the exception that amino acid-free RPMI (United States Biological, Life Sciences, Salem, MA) supplemented with glutamine, isoleucine and methionine (300 mg/L, 50 mg/L, and 15 mg/L, respectively, Sigma-Aldrich, St Louis, MO) was mixed with complete RPMI in a 3:1 ratio to make amino acid-limited media.

### Design and construction of CRISPR-Cas9 plasmid vectors for transfection

Transfections were performed using a three-plasmid strategy. The first plasmid contained homology regions (HRs) to the gene of interest (GOI) flanking a *hdhfr* positive selectable marker, while the other plasmids contained the coding sequence for Cas9 and a single guide RNA (gRNA) specific to each GOI, targeting two sites in the GOI in total.

To generate the basic template vector pGEM*hdhfr* + , the *hdhfr* positive selectable marker cassette was PCR amplified from the vector pL-6_eGFP [87] with primers *hdhfr* 5’ F and *hdhfr* 3’ R and cloned into pGEM-3Z (Promega, Madison, WI) digested with *Hinc*II. Two flanking regions corresponding to the GOI were then cloned into this basic vector to generate a template for homologous recombination and disruption of the GOI. The HRs were amplified from 3D7 gDNA with the following primer sets from Liu *et al*., [34] - *plasmepsin II* 5’ HR: PMII 5’ HR Fwd and PMII 5’ HR Rev; *plasmepsin II* 3’ HR: PMII 3’ HR Fwd and PMII 3’ HR Rev; *plasmepsin III* 5’ HR: PMIII 5’ HR Fwd and PMIII 5’ HR Rev; and *plasmepsin III* 3’ HR: PMIII 3’ HR Fwd and PMIII 3’ HR Rev. The 3’ HRs were digested with *Pst*I and *Sph*I and ligated into the appropriately digested pGEM*hdfr+* basic template vector. The coordinating 5’ HRs were digested with *Afl*II and *Xma*I and ligated into the pGEM*hdfr+* vector already containing the 3’ HR. The *plasmepsin II/III* double KO construct contains a *plasmepsin II* 5’ HR and a *plasmepsin III* 3’ HR flanking the *hdhfr* cassette. The HR plasmids were transfected into parasites in either their circular or *Bgl*I-linearized form. GOI-specific gRNA sequences were generated using Benchling (https://benchling.com) and ligated into the *Bbs*I digested pDC2-Cas9-U6-hDHFR vector [88]. All primers used are described in S11 Table.

### Parasite transfection and selection

Parental KH001_053_G8_ and KH001_053_G10_ parasites were synchronized using 5% D-sorbitol (Sigma-Aldrich, St. Louis, MO) and early ring-stage parasites were mixed with 50 μg of each transfection plasmid in cytomix (120 mM KCl, 0.15 mM CaCl2, 2 mM EGTA, 5 mM MgCl2, 10 mM K2HPO4/KH2PO4 (pH 7.6), 25 mM HEPES (pH 7.6) and electroporated in 2 mm cuvettes at 0.31 kV and 950 μF [89]. Electroporated cells were then placed in O^+^ media with fresh erythrocytes at 5% hematocrit. After 24–48 h of drug-free recovery, cultures were continuously treated with 5 nM WR99210, which positively selects for parasites containing *hdhfr*. Recovered parasites were genotyped as bulk cultures and subcloned by limiting dilution. Subsequently, clonal lines were genotyped by PCR, qPCR, and Southern blotting. All primers used are described in S11 Table.

### Polymerase chain reaction (PCR) for genotyping transfectants

Genomic DNA was isolated from bulk transfectant cultures and from the resulting clonal lines using the QIAamp Blood Mini Kit (Qiagen, Hilden, Germany). To detect the presence of *hdhfr* integration at the GOI, PCR was performed upon isolated gDNA using the Phusion High-Fidelity PCR Mastermix (New England BioLabs, Ipswich, MA), according to the manufacturer’s instructions. For screening purposes, PCR reactions were also performed direct-from-culture, using a 1:100 dilution of parasitized packed human erythrocytes in the final PCR reaction.

Parasites that were wildtype at the *plasmepsin II* locus were detected using primers located upstream and downstream of the gene (PM2 5’ Upstream Fwd and PM2 3’ Downstream Rev), respectively. Integration of the *hdhfr* cassette at the *plasmepsin II* locus was detected using a forward primer upstream of the gene and a reverse primer located in the *hdhfr* cassette (PM2 5’ Upstream Fwd and *hdhfr* cassette 5’ Rev). To detect parasites that were wildtype at the *plasmepsin III* locus, a forward primer specific for the sequence between the homology regions and a reverse primer located downstream of the gene (PM3 Btwn HRs Fwd and PM3 3’ Downstream Rev) were used. Integration of the *hdhfr* cassette at the *plasmepsin III* locus was detected using a forward primer found in the *hdhfr* cassette and a reverse primer located downstream of the gene (*hdhfr* cassette 3’ Fwd and PM3 3’ Downstream Rev). All *plasmepsin II/III* KO transfectants were probed for integration at both the *plasmepsin II* and **plasmepsin* III* loci. The presence of plasmid was probed using primers specific to the backbone of the pGEM*hdhfr+* template vector (pGEM_hdhfr+ Fwd and pGEM_hdhfr+ Rev) for all transfections. All primer sequences are listed in S11 Table.

### Quantitative PCR

To determine copy numbers of *pfmdr*1, **plasmepsin* II*, and *plasmepsin III*, qPCR was performed on genomic DNA (extracted with QIAmp Blood Mini Kit, Qiagen, Hilden, Germany) as previously described [29] with the following modifications: amplification reactions were done in MicroAmp 384-well plates in 10 μl SYBR Green master mix (Applied Biosystems, Foster City, CA), 150 nM of each forward and reverse primer and 0.4 ng template. Forty cycles were performed in the Applied Biosystems ViiATM 7 Real-time PCR system (Life Technologies, Carlsbad, CA). *pfmdr1* primers were designed after Price *et al*.[90] whereas *β- tubulin* primers for the endogenous control were designed after Ribacke *et al*.[91]. To test for integration of the *hdhfr* cassette into the *plasmepsin* locus, two primer sets were designed located between the HRs for *plasmepsin II* (RTPCR PMII forw and rev) and *III* (RTPCR PMIII forw and rev) as well as on the *hdhfr* gene (*hdhfr*_RTPCR_F and R). Technical replicates were run in quadruplicate. Copy numbers were considered increased (>1) when the average of three biological replicates was above 1.6. Primers are listed in S11 Table.

### Southern blotting

Parasite-infected red blood cells were lysed with 0.15% saponin (Sigma-Aldrich, St. Louis, MO) in PBS and gDNA was isolated from freed parasites via phenol-chloroform extraction. For each clone, 5 ug gDNA was digested with the following restriction enzymes in the KH001_053_G10_ lines: *Eco*O1091 and *Xmn*I for the *plasmepsin II* KO clones; *Kpn*I and *Nsi*I for the *plasmepsin III* KO clones; *Eco*O1091, *Xmn*I, *Kpn*I and *Nsi*I for the *plasmepsin II/III* double KO clones; *Afl*II and *Nci*l for the *plasmepsin II* KO and *plasmepsin III* KO clones in a KH001_053_G8_ parental background and all *plasmepsin II/III* KO clones. As controls, gDNA from the parental lines (KH001_053_G8_ and KH001_053_G10_) and corresponding plasmid vectors were also digested. Digested gDNA was resolved on a 1% agarose gel in TAE and transferred to Amersham Hybond – N^+^ nylon transfer membrane (GE Healthcare, Chicago, IL). Probe hybridization and horseradish peroxidase-mediated signal detection were performed using the ECL Direct Nucleic Acid Labeling and Detection System (GE Healthcare, Chicago, IL), following the manufacturer’s instructions. DNA probes were designed to detect *hdhfr*, *plasmepsin II* 3’ HR, or **plasmepsin* III* 3’ HR. The *hdhfr* probe was PCR amplified from plasmid vector, while the *plasmepsin II* 3’ HR and *plasmepsin III* 3’ HR probes were PCR amplified from KH001_053_G10_ gDNA; the corresponding primers are listed in S11 Table.

### *In vitro* drug susceptibility assays by SYBR Green I staining

Drug susceptibility assays were performed using the SYBR Green I method as previously described [92]. In brief, tightly synchronized 0–6 h post-invasion rings (by a Percoll gradient and D-sorbitol synchronization 6 h later, Sigma-Aldrich, St. Louis, MO) at 1% parasitemia and 1% hematocrit in 40 uL of 0.5% Albumax-complemented RPMI media were grown for 84 h in 384-well clear bottom plates, in the presence of different drug concentrations. Drug assays were extended from the standard 72 h drug exposure to 84 h due to the tight synchronization of the parasites. All drug conditions were performed in three technical replicates, with at least three biological replicates. Drugs were dispensed in 24-point dilution series of PPQ (0.07 nM–50 μM) and 12-point dilution series of all other drugs (CQ, E64, MQ, DHA, WR99210, pepstatin A methyl ester (pepA), and AQ; Sigma-Aldrich, St. Louis, MO) into 384-well plates by an HP D300e Digital Dispenser (Hewlett Packard, Palo Alto, CA). Growth at 84 h was quantified by staining parasite DNA with SYBR Green I (Lonza, Visp, Switzerland) for 24 h, and measuring relative fluorescence units at an excitation of 494 nm and an emission of 530 nm using a SpectraMax M5 (Molecular Devices, San Jose, CA). Half-maximal effective concentration (EC_50_) values, for all drugs except PPQ, were calculated using a nonlinear regression with the log(inhibitor) vs. response-Variable slope curve-fitting algorithm using GraphPad Prism version 8–10 (GraphPad Software, La Jolla, CA). PPQ susceptibility was quantified using area under the curve (AUC) between the two local minima of the high-dose peak, as previously described [29].

PPQ stocks were resuspended in a 0.5% lactic acid/0.1% Triton X-100 aqueous solution, CQ stocks were resuspended in a 0.1% Triton X-100 aqueous solution, and all other drugs were resuspended in dimethyl sulfoxide (DMSO).

For drug susceptibility assays performed in amino acid-limited conditions, amino acid-limited media was generated as described above and parasites were transferred to amino acid-limited media right before the start of the drug assay.

To test the effects of DV stress in modulating PPQ response, parasites were treated with a 24-point dilution series of PPQ in combination with a fixed concentration of the additional inhibitor (either E64: 2.6 μM or 3.9 μM; pepstatin A methyl ester (pepA): 5 μM or 7.5 μM; CCCP: 5 μM or 15 μM; concanamycin A (ConA): 0.1 nM or 0.2 nM; or DMSO control (Sigma-Aldrich, St. Louis, MO). Relative growth was normalized in comparison to parasite growth in the presence of E64, pepA, CCCP, or ConA at the test concentration alone.

To determine the role of pH in drug susceptibility, parasites were exposed to a dilution series of PPQ or CQ in pH-adjusted Albumax-complemented media for 84 h at pH values of ~6.75, ~ 7.5, or ~8.25.

### Cellular heme fractionation assay

Baseline levels of different heme species in the parasite lines were determined using pyridine-based detergent-mediated cellular heme fractionation assays described in detail by Combrinck *et al*. [44,45] For the drug exposure experiments with KH001_053_G10_ and KH001_053_G8_, parasites were synchronized to ring stages using two to three cycles of D-sorbitol treatment and early rings were incubated at 37˚C at 5% parasitemia and 2% hematocrit in 24-well plates. After 32 h, late trophozoites were harvested by lysing red blood cells with 0.05% saponin followed by multiple washes with 1 × PBS (pH 7.5). Pellets were then resuspended in 1 × PBS and stored at −80°C before further analysis. An aliquot of the trophozoite suspension was stained by SYBR Green I and quantified via flow cytometry (described in detail below) to determine the total number of trophozoites. For determining the heme composition throughout the life cycle, we increased the starting culture volume to 10 mL of 5% parasitemia at 2.5% hematocrit and further tightened the synchronization to 0–6 h post-erythrocyte-invasion rings by a Percoll gradient and D-sorbitol synchronization 6 h later. Parasites were harvested at 24, 36 and 42 h post D-sorbitol synchronization. One set of parasites was exposed to 2 μM PPQ between 24 and 36 h and harvested at 36 h.

Samples were thawed and DV content released from trophozoites by hypotonic lysis and sonication (53 kHz, 320 W). Parasite fractions corresponding to digested hemoglobin, free heme-Fe, and hemozoin were then carefully recovered through centrifugation and treatment with HEPES buffer (pH 7.4), 4% SDS, 25% pyridine solution, 0.3 M HCl and 0.3 M NaOH (Sigma-Aldrich, St. Louis, MO).

The UV-visible spectrum of each heme fraction was measured as a Fe^3+^-heme-pyridine complex using a multi-well SpectraMax M5 plate reader (Molecular Devices, San Jose, CA). The total amount of each heme-Fe species in a sample was quantified using a heme standard curve and interpolation. The percentage of each species per sample was compared between the different strains and conditions. Two-tailed unpaired Student’s t-tests were used for comparing PPQ-treated vs untreated lines at the same time point or between KH001_053_G10_ and the other strains.

### Flow cytometry to quantify parasitemia and stage

Parasites were stained in 10X SYBR Green I in 1xPBS for 30 min in the dark at 37°C. The staining solution was removed, and cells were resuspended in five times the volume of the initial volume of PBS. Flow cytometry data acquisition was performed on a MACSQuant VYB (Miltenyi Biotec) with a 488 nm laser and a 525 nm filter and analyzed with FlowJo 2. Red blood cells were gated on the forward light scatter and side scatter and infected red blood cells were detected in channel B1. At least 100,000 events were analyzed per sample. Parasites were considered trophozoites when the DNA content was 3 times higher than the ring-stage signal.

### Metabolite extraction for HPLC-MS data collection

Synchronized cultures of trophozoite stage (24–28 h post-invasion) parasites (5–10% parasitemia, 2% hematocrit) were purified from uninfected red blood cells by VarioMacs Magnet using a MACS CS column (Miltenyi Biotec. Charlestown, MA). The purified parasite pellet was resuspended at 5x10^7^ – 1x10^8^ cells/mL in Albumax media, and allowed to recover for 1 h at 37°C. For comparison between R7 clones B9 and D4, parasites were processed directly. For drug exposure experiments, following recovery, purified trophozoites were incubated in 6-well plates with either 10 nM atovaquone (ATQ, positive control), 140 nM PPQ, or no-drug control for 2.5 h at 37°C. Immediately following treatment, parasite pellets were washed with 1 mL 1x ice-cold PBS, before being resuspended in 1 mL prechilled 90:10 methanol-water and placed on ice. Samples were vortexed, resuspended and centrifuged for 10 min at 15,000 rpm and 4°C. Samples were stored at −80°C before being dried down under nitrogen flow for UHPLC-MS analysis. The dried metabolites were resuspended in HPLC-grade water (Chromasolv; Sigma-Aldrich, St. Louis, MO), containing chlorpropamide as an internal control, to a concentration between 1x10^5^ and 1x10^6^ cells/mL, based on hemocytometer counts of purified parasites.

### HPLC-MS data collection

All samples were processed in triplicate with method blanks to reduce technical variation and account for background signal. Samples were randomized with pooled quality control samples and blanks run regularly throughout the runs.

For untargeted putative-hemoglobin peptide analysis, 5 μL of each sample was injected for analysis. Metabolites were separated using a reversed phase method on a HPLC Prominence 20 UFLCXR system (Shimadzu, Marlborough, MA) using a Waters BEH C18 column (100 mm x 2.1 mm 1.7 μm particle size) at 55 °C and an aqueous acetonitrile gradient run for 20 min at a flow rate of 250 μL/min. Solvent A was HPLC grade water with 0.1% formic acid, and Solvent B was HPLC grade acetonitrile with 0.1% formic acid. The solvent gradient was at 0.0 min 3% of B, 10.0 min 45% of B, 12.0 min 75% of B, 17.5 min 75% of B, and 18.0–20.0 min: 3% of B. Eluate was delivered into a (QTOF) 5600 TripleTOF using a DuoSprayTM ion source (AB Sciex, Toronto Canada). Capillary voltage was 5.5 kV in positive and 3.8 kV in negative ion mode with declustering potentials of 80V and −80V respectively. The TripleTOF was scanning 50–1000 m/z, and 16 MS/MS product ion scans (100 ms) per duty cycle using collision energy of 50V with a 20V spread.

For targeted metabolomics analysis, 10 µL of each sample was injected for analysis. For each sample, 10 µL were injected through a XSelect HSS T3 2.5 µM C18 column (Waters, Millford, MA #186006151) at 30˚ C and eluted using a 200 µL/min 25 min gradient of 3% aqueous methanol, 15 mM acetic acid (Millipore Sigma, Burlington, MA, #A6283), 10 mM tributylamine (Millipore Sigma, Burlington, MA, #90781), 2.5 µM medronic acid ion pairing agent (Millipore Sigma, Burlington, MA, #M9508) (A) and 100% HPLC-grade methanol (B), with the gradients: 0–5.0 min 100% A, 0% B; 5.0–13.0 min 80% A, 20% B; 13.0–15.0 min 45% A, 55% B; 15.0–17.5 min 35% A, 65% B; 17.5–21.0 min 5% A, 95% B; 21.0–25 min 100% A, 0% B. Negative-ion mode, using a scan range of 85–1,000 *m/z* and a resolution of 140,000 at *m/z* 200 was used for ion detection.

### HPLC-MS data processing

MSConvert of the ProteoWizard software package was used to convert the.wiff/.wiffscan (untargeted) or.raw (targeted) data files to.mzML formatted files compatible with MS-Dial or El-Maven, which were used to align, group, quantify and initially visualize the untargeted and targeted data, respectively. For putative hemoglobin-derived peptides analysis using untargeted data, peak areas from both positive and negative modes were exported, and the resulting feature quantification matrices were used as input for a custom R script designed to putatively annotate peak groups as human hemoglobin-derived peptides of matching m/z value within 15 ppm, considering all potential human hemoglobin peptides up to 13 amino acids long. The resulting table of annotated peptides was then exported to Microsoft Excel for final processing. For targeted analysis, feature quantification matrices were exported directly from El-Maven to Microsoft Excel for final processing.

For both datasets, within each biological replicate, the chlorpropamide control signal was used as an internal control for instrument variation correction. The blank signals were then subtracted from the chlorpropamide-corrected data, and a detection reproducibility filter was applied to the list of metabolites to exclude those that had a relative standard deviation across the pooled quality control samples of greater than 30. Log_2_ fold changes were calculated relative to no-drug controls, and the resulting values across biological replicates were used to generate volcano plots using GraphPad Prism 10.

All metabolomics data has been made publicly available through the Metabolomics Workbench (https://www.metabolomicsworkbench.org, NIH grant U2C-DK119886) [93] with the following study numbers: ST003904, ST003902, and ST003906.

### Digestive vacuole pH determination

Saponin-isolated trophozoite-stage parasites containing the membrane-impermeant pH-sensitive fluorescent indicator fluorescein-dextran (10,000 MW; Invitrogen, Carlsbad, CA) in their DVs were prepared as outlined previously [55,58]. The isolated parasites were washed and suspended in malaria saline (125 mM NaCl, 5 mM KCl, 1 mM MgCl_2_, 20 mM glucose, 25 mM HEPES; pH 7.1) at a density of ~2–3 × 10^7^ cells/mL. Isolated parasite suspensions were checked for integrity pre- and post-experiment by light microscopy of Giemsa-stained parasites. Prior studies have demonstrated that parasites isolated in this manner maintain ion gradients and membrane potentials for >3 h under the conditions tested here [94–96]. 100 µL of cell suspension was added to an equal volume of malaria saline containing test compounds at 2x test concentrations in a 96-well clear-bottomed black plate. The pH of the DV was monitored at 37^°^C over 1.5 h using a Molecular Devices M5i plate reader (excitation 490 and 450 nm, emission 520 nm). Fluorescence ratios (490/450) were calibrated to pH units using calibration buffers at pH 4.5, 5.1, 5.7 and 6.3 (130 mM KCl, 1 mM MgCl_2_, 20 mM glucose, 25 mM HEPES, 180 nM nigericin, 100 nM ConA) as described previously [55,58].

## Supporting information

S1 Fig Confirmation of *plasmepsin II* and *III* single KOs clones by Southern blot. (A) Schema of original loci, homology plasmids used for integration into the locus and resulting edited loci. Restriction enzyme sites and expected band sizes for Southern blots are indicated in the schema in orange for *plasmepsin II* and in blue for *plasmepsin III* KOs. (B) Southern blots with different probes, expected band sizes are indicated by arrows. Clones indicated with a star were used for phenotyping. Plasmids were either transfected in circular (cir) or linearized (lin) form. (C) WR99210 primarily targets the plasmodial *dhfr* and an increase in EC_50_ is correlated with the presence of one or several *hdhfr* cassettes present. Shown is the average EC_50_ and standard deviations of three biological replicates for each clone (one-way ANOVA with Dunnett’s post-test compared to 1D with a single integration of the *hdhfr* cassette. ****p < 0.0001).
(TIF)

S2 Fig Confirmation of *plasmepsin II*/*III* double KOs clones by Southern blot. (A) Schema of original parental loci, homology plasmid used for integration into the loci, and resulting edited locus which is identical for both parents. Restriction enzyme sites and expected band sizes for Southern blots are indicated in the schema in orange for *plasmepsin II* and in blue for *plasmepsin III* KOs. (B) Southern blots with *plasmepsin III* probe, expected band sizes are indicated by arrows. Clones indicated with a star were used for phenotyping. (C) Average EC_50_ and standard deviations of three biological replicates of WR99210 for each clone except 4E (n = 1). nd: not determined.
(TIF)

S3 Fig Clonal *plasmepsin II/III* double KO lines from single locus and double locus parents are indistinguishable by Southern blot. (A) Schema of original parental loci, homology plasmid used for integration into the loci and resulting edited locus which is identical for both parents. Restriction enzyme sites outside the locus were selected to confirm complete deletion of the regions between the homology regions and expected band sizes for Southern blots are indicated in the schema. Clones indicated with a star were used for phenotyping. (B) The same Southern blots was hybridized three times with the *plasmepsin II*, *plasmepsin III*, or *hdhfr* probe. Expected band size for each probe is indicated with arrows. The loss of hybridization for the *plasmepsin II* probe confirms the deletion and fusion of *plasmepsin II* and *plasmepsin III* in the KO clones.
(TIF)

S4 Fig Parasite growth in different concentrations of drug compared to no drug control. Ring-stage parasites were either grown in complete media or in complete media with the addition of CCCP (15 or 5 μM), E64 (3.9 or 2.6 μM), pepA (7.5 or 5 μM), ConA (0.1 or 0.2 nM), 10 μM DHA (dead) or 0.5% DMSO for 72 h. Growth was measured by the incorporation of SYBRGreen into DNA, read by a spectrometer and normalized to parasites cultured in media only.
(TIF)

S5 Fig Effect of protease inhibitors on PPQ efficacy in parasites with a single (KH001_053_G10_), double (KH001_053_G8_) or multiple (KH004_057) *plasmepsin* loci. Parasites were exposed to increasing levels of PPQ in the presence of DMSO or (A) E64 at a concentration of either 2.6 μM or 3.9 μM or (B) pepstatin A (pepA) at a concentration of either 5 μM or 7.5 μM. Shown is one example of three biologically independent experiments run in triplicates. (C) Average and SD of the area under the curve (AUC) between the local minima for three biological replicates. No statistically significant difference was detected between PPQ alone and PPQ in combination with either E64 or pepA by ordinary one-way ANOVA with Tukey post-test.
(TIF)

S6 Fig PPQ does not inhibit hemoglobin-derived peptide formation. A and B: Two clones from the Cambodian RF7 parasite line [22] with either one copy (B9) or three copies (D4) of *plasmepsin II* and *III* were used for small molecule metabolomic analysis, which includes relative quantitation of short peptides. Metabolomic analysis was run in both positive and negative mode and a total of 35 putative endogenous hemoglobin-derived peptides (*i.e.*, dipeptides to 13-mers) were detected based on their m/z match that could be mapped to either the alpha (A) or beta (B) chains of hemoglobin. Shown are the volcano plots combining statistical significance and fold change observed in metabolites from RF7 clones D4 compared to B9. C to F: Effects of PPQ or ATQ treatment on the parasite’s metabolism. Purified *P. falciparum* 3D7 trophozoites were treated for 2.5 h with 140 nM PPQ or 10 nM ATQ (as a control) and volcano plots comparing metabolites from untreated vs PPQ-treated parasites for targeted metabolite analysis from PPQ (C) and ATQ (D) treated parasites compared to untreated are shown. Volcano plots comparing metabolites from untargeted analysis of all putative hemoglobin-derived peptides of amino acid length 13 or less are shown in (E) (alpha chain) and (F) (beta chain). The dotted lines depict the significance cutoff of *p* = 0.01 and a two-fold change in metabolite abundance. Only in N-carbamoyl-L-aspartate and dihydroorotate under ATQ treatment were significantly increased in abundance [46].
(TIF)

S7 Fig Growth is dependent on amino acid concentration. We determined the minimal amino acid needs of parasites to allow for enough DNA replication to perform drug susceptibility assays by SYBR Green I staining. KH001_053_G10_, KH001_053_G8_, G8_PMII/III_KO_ and KH004_057 were synchronized and set up at 1% parasitemia and 2% hematocrit in regular RPMI media or RPMI media with isoleucine, methionine, and glutamine as the only amino acid sources. The two conditions were then mixed in 10% increments (90% regular media plus 10% amino acid-free (except isoleucine, methionine, and glutamine) media, 80% and 20% etc.) in 96 well plates and incubated at 37°C for 72 h. Growth was analyzed by adding SYBR Green to the plates, measuring the fluorescence, and normalizing the signal to parasites grown in regular media. The final assays conditions for drug susceptibility assays were set at 25% full amino acid RPMI and termed amino acid-limited media.
(TIF)

S8 Fig Parasite lines are similarly affected by low amino acid concentrations. KH001_053_G10_, KH001_053_G8_, G8_PMII/III_KO,_ and KH004_057 parasites were synchronized and set up at 0.5% parasitemia in either regular media or amino acid-limited media. Parasite replication was measured by estimating the parasitemia in the second cycle by flow cytometry of SYBR Green-stained parasite samples and dividing it by the initial parasitemia. Shown are the average replication rates for three biological replicates with SD. There were no statistically significant differences detected between the strain grown in either regular or amino acid-limited media by one-way ANOVA followed by Dunnett’s post-test, ns = no significance. The replication rate for all strains was significantly less in amino acid-limited media compared to regular media by Student’s t-test: *p < 0.05.
(TIF)

S9 Fig Effect of internal or external pH on CQ efficacy. A-F) Parasites were exposed to increasing levels of CQ in acidic (pH = 6.74), normal (pH = 7.5) or basic (pH = 8.24) media. Shown is one example for each tested line of three biologically independent experiments run in triplicates. G-L) Parasites were exposed to increasing levels of CQ in the presence of DMSO or CCCP at a concentration of either 5 μM or 15 μM. The EC_50_ was calculated where possible (an # indicates when parasites were not killed completely at the highest concentration), and the average and SD are shown in (F and L). Statistics show one-way ANOVA with Tukey post-test for each strain tested in the presence of CCCP or two tailed paired Student’s t-test for the external pH changes: *p < 0.05; **p < 0.01; ***p < 0.001.
(TIF)

S1 Table Analysis of parasite clones from different transfections to confirm proper integration into the genome by PCR and qPCR. Plasmids were either transfected in circular form (cir) or were linearized with *Bgl*I before transfection (lin). Shown are all the clones analyzed for every transfection that was recovered. Integration was confirmed by PCR of the 5’ and 3’ region and appearance of a band at the right size was considered a positive result (green check mark). The absence of the transfection plasmid was screened for with primers targeting the backbone of the plasmid which is lost after correct integration (red cross). Quantitative PCR was also used to confirm copy numbers of *plasmepsin II* and *III* as well as the *hdhfr* gene inserted into the locus. Primers are listed in S11 Table.
(XLSX)

S2 Table EC_50_ data for various drugs tested. Parasite lines were exposed to various concentration of drug and the EC_50_ was calculated for each drug. Each experiment was run in triplicate and at least three biological replicates were performed for each parasite line/clone. Shown are the EC_50_, SD and sample size for each line.
(XLSX)

S3 Table Combination assays results and amino acid-limited conditions (Fig 5, 6E–L and S9 Fig). (A) Combination assays of parasites in the presence of increasing concentrations of PPQ and a constant concentration of E64, pepA, ConA, or CCCP measured as AUC. (B) Parasites were cultured either in regular or amino acid-limited media and exposed to increasing concentrations of PPQ and the AUC was measured. (C) EC_50_ for ConA and CCCP. (D) Combination assays in the presence of increasing concentrations of CQ and a constant concentration of CCCP measured as EC_50_.
(XLSX)

S4 Table Data for heme fractionation of PPQ-treated and untreated parasites with variable *plasmepsin* copy numbers (Fig 3A–C). Different heme species were extracted from parasites by subsequent cellular fractionation steps. Tightly synchronized KH001_053_G10_ and KH001_053_G8_ ring-stage parasites were exposed to various PPQ concentrations for 32 h. The amount of iron species (Fe) per sample was estimated based on the standard curve run for each biological replicate and the percentages for each species per sample were calculated. Shown is each value from three biological replicates run in quadruplicate with the average and SD of percentage of hemozoin Fe, hemoglobin, and free heme Fe. Statistical comparisons of the drug-treated lines to their untreated controls were performed using two-tailed unpaired Student’s t-tests *p < 0.05; **p < 0.01.
(XLSX)

S5 Table Data for heme fractionation of *plasmepsin* KO parasites throughout the life cycle and exposed to PPQ (Fig 3D–F and Fig 4). Tightly synchronized parasites were harvested at different time-points throughout the life cycle; the average and SD of percentage of hemozoin Fe, hemoglobin, and free heme Fe are shown for three independent experiments for parasites with a single locus (KH001_053_G10_), duplicated locus (KH001_053_G8_), G8_PMII_KO_, G8_PMIII_KO_, or G8_PMII/III_KO_. Statistical comparisons at each time point were performed between the single copy KH001_053_G10_ parasites and all other lines using two-tailed unpaired Student’s t-tests *p < 0.05; **p < 0.01. Additionally, parasites were incubated with 2 μM PPQ from 24 to 36 h post synchronization and harvested at 36 h. Statistical comparisons of PPQ-treated to untreated parasites at the 36 h timepoint from three independent biological replicates were performed using two-tailed unpaired Student’s t-tests *p < 0.05; **p < 0.01; ***p < 0.001.
(XLSX)

S6 Table Data for untargeted metabolomics of hemoglobin peptides of parasites with different *plasmepsin* copy numbers. Two clones from the Cambodian RF7 parasite line with either one copy (B9) or three copies (D4) of *plasmepsin II* and *III* were used for metabolomic analysis, which includes the relative quantitation of short peptides [22]. High performance liquid chromatography-mass spectrometry (HPLC-MS)-based metabolomic analysis was run in both positive and negative mode, and a total of 35 putative endogenous hemoglobin derived peptides (dipeptides to 13-mers) based on m/z match were detected that could be mapped to either the alpha or beta chains of hemoglobin. The log_2_ fold changes of D4/B9 of all detected hemoglobin peptides are shown for three biological replicates each by positive and negative mode. The combined average log_2_ of D4/B9 and the -log_10_(p-value) of all putative peptides were plotted in S6A and S6B Fig.
(XLSX)

S7 Table Data for targeted metabolomics of hemoglobin peptides of PPQ- and ATQ-treated parasites. 3D7 trophozoites were treated for 2.5 h with 140 nM PPQ or 10 nM ATQ and the log_2_ fold changes for PPQ/no drug and ATQ/no drug are listed for three and six independent biological replicates, respectively. Data for this targeted approach is collected in negative mode and uses a retention-time-validated reference set of 115 targeted metabolites [46]. Only in N-carbamoyl-L-aspartate and dihydroorotate under ATQ treatment were significantly upregulated, as expected from previous studies [46]. The combined average log_2_ of PPQ/no drug and ATQ/no drug and the -log_10_(p-value) of all samples were plotted in S6C and S6D Fig.
(XLSX)

S8 Table Data for untargeted metabolomics of hemoglobin peptides of PPQ-treated parasites. 3D7 trophozoites were treated for 2.5 h with 140 nM PPQ and the log_2_ fold changes for PPQ/no drug are listed for three independent biological replicates. High performance liquid chromatography-mass spectrometry (HPLC-MS)-based metabolomic analysis was run in both positive and negative mode and a total of 220 putative endogenous hemoglobin derived peptides (dipeptides to 13-mers) were detected based on m/z match that could be mapped to either the alpha or beta chains of hemoglobin. The combined average log_2_ of PPQ/no drug and the -log_10_(p-value) of all samples were plotted in S6E and S6F Fig.
(XLSX)

S9 Table Effect of extracellular pH on drug response (Fig 6A and S9 Fig). Parasites were exposed to increasing levels of CQ or DHA for 72 h or PPQ for 84 h in acidic (pH = 6.74), normal (pH = 7.5) or basic (pH = 8.24) media. Included are the EC_50_ or AUC data for four biologically independent experiments run in triplicates for Dd2, 3D7, KH001_053_G10_, KH001_053_G8,_ and KH004_057 including the average and SD of the AUC or EC_50_. Unpaired Student’s t-test between pH at 7.5 and lower or higher pH if more than three values could be determined: **p < 0.01, ***p < 0.001.
(XLSX)

S10 Table Digestive vacuole pH measurement of fluorescein-dextran loaded Dd2 parasites. A) DV pH traces of Dd2 parasites exposed to PPQ (50 μM, 10 μM, 5 μM and 1 μM) and control treatments Concanamycin A (ConA, 100 nM), CCCP (10 µM), CQ (10 µM) and NH_4_Cl (10 mM) over 90 min run in technical duplicates. B) DV pH was quantified for each treatment as an average of the measurements taken between 45 and 60 mins of compound exposure. Shown is the average pH for three to four independent experiments (performed with blood from different donors).
(XLSX)

S11 Table List of primers used. This table includes all primers used in this study.
(XLSX)
