# Supplementary figures and images for: The plasmepsin-piperaquine paradox persists in Plasmodium falciparum
Source: PLoS Pathog. 2025 Jul 28;21(7):e1012779. doi: 10.1371/journal.ppat.1012779 (PMC12321132; doi:10.1371/journal.ppat.1012779)

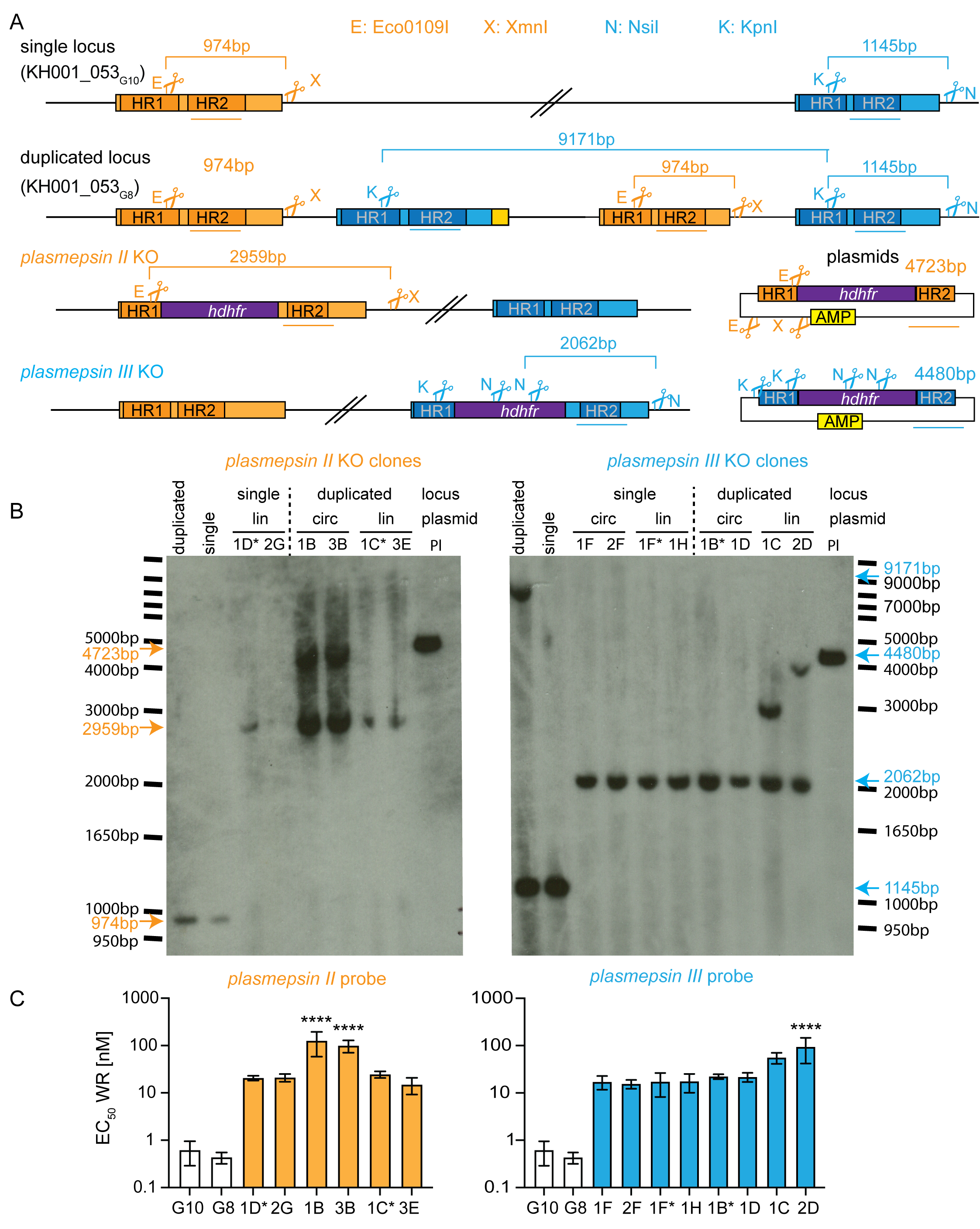

Supplement: S1 Fig — (A) Schema of original loci, homology plasmids used for integration into the locus and resulting edited loci. Restriction enzyme sites and expected band sizes for Southern blots are indicated in the schema in orange for plasmepsin II and in blue for plasmepsin III KOs. (B) Southern blots with different probes, expected band sizes are indicated by arrows. Clones indicated with a star were used for phenotyping. Plasmids were either transfected in circular (cir) or linearized (lin) form. (C) WR99210 primarily targets the plasmodial dhfr and an increase in EC50 is correlated with the presence of one or several hdhfr cassettes present. Shown is the average EC50 and standard deviations of three biological replicates for each clone (one-way ANOVA with Dunnett’s post-test compared to 1D with a single integration of the hdhfr cassette. ****p < 0.0001). (TIF) [file ppat.1012779.s001.tif]

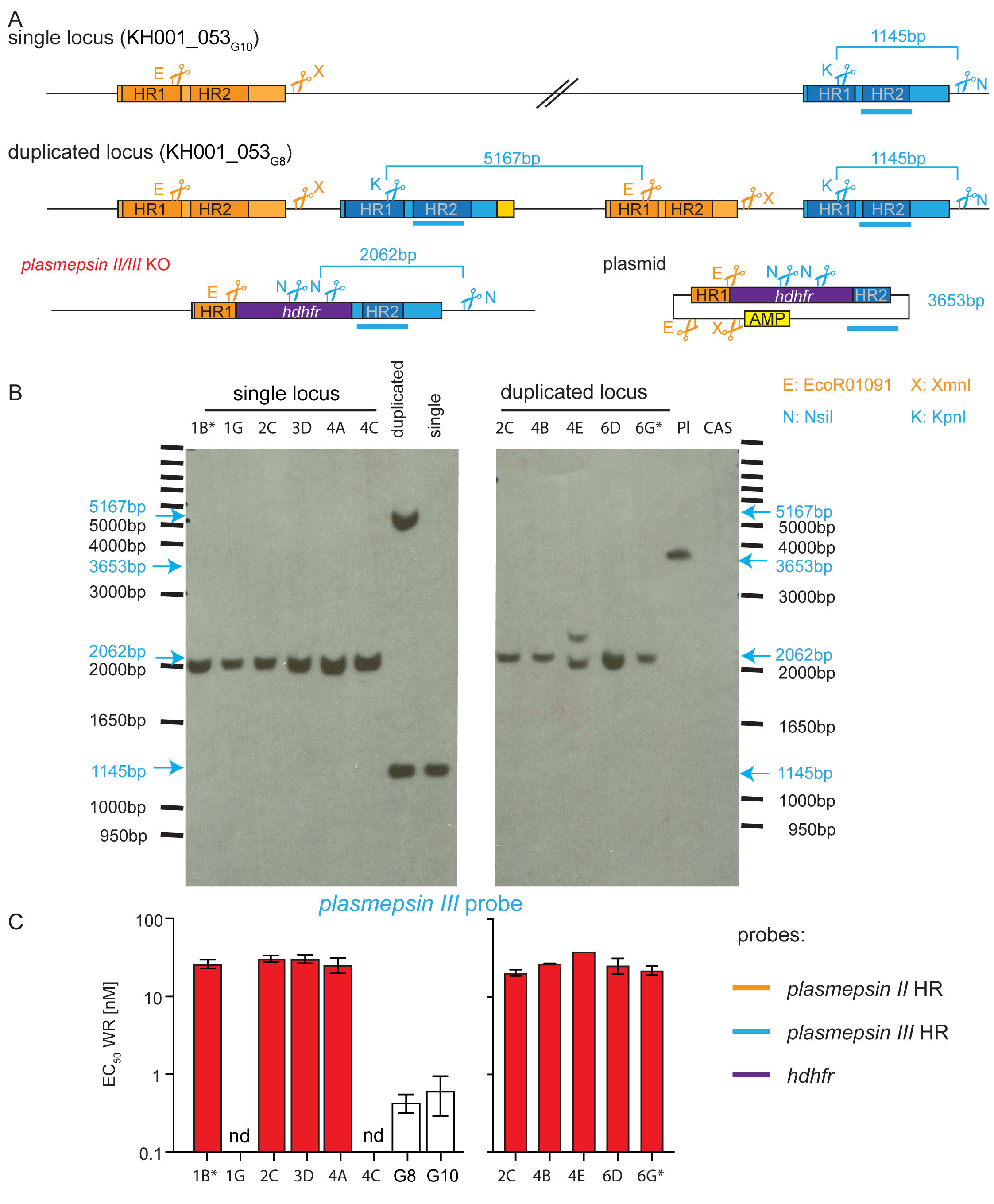

Supplement: S2 Fig — (A) Schema of original parental loci, homology plasmid used for integration into the loci, and resulting edited locus which is identical for both parents. Restriction enzyme sites and expected band sizes for Southern blots are indicated in the schema in orange for plasmepsin II and in blue for plasmepsin III KOs. (B) Southern blots with plasmepsin III probe, expected band sizes are indicated by arrows. Clones indicated with a star were used for phenotyping. (C) Average EC50 and standard deviations of three biological replicates of WR99210 for each clone except 4E (n = 1). nd: not determined. (TIF) [file ppat.1012779.s002.tif]

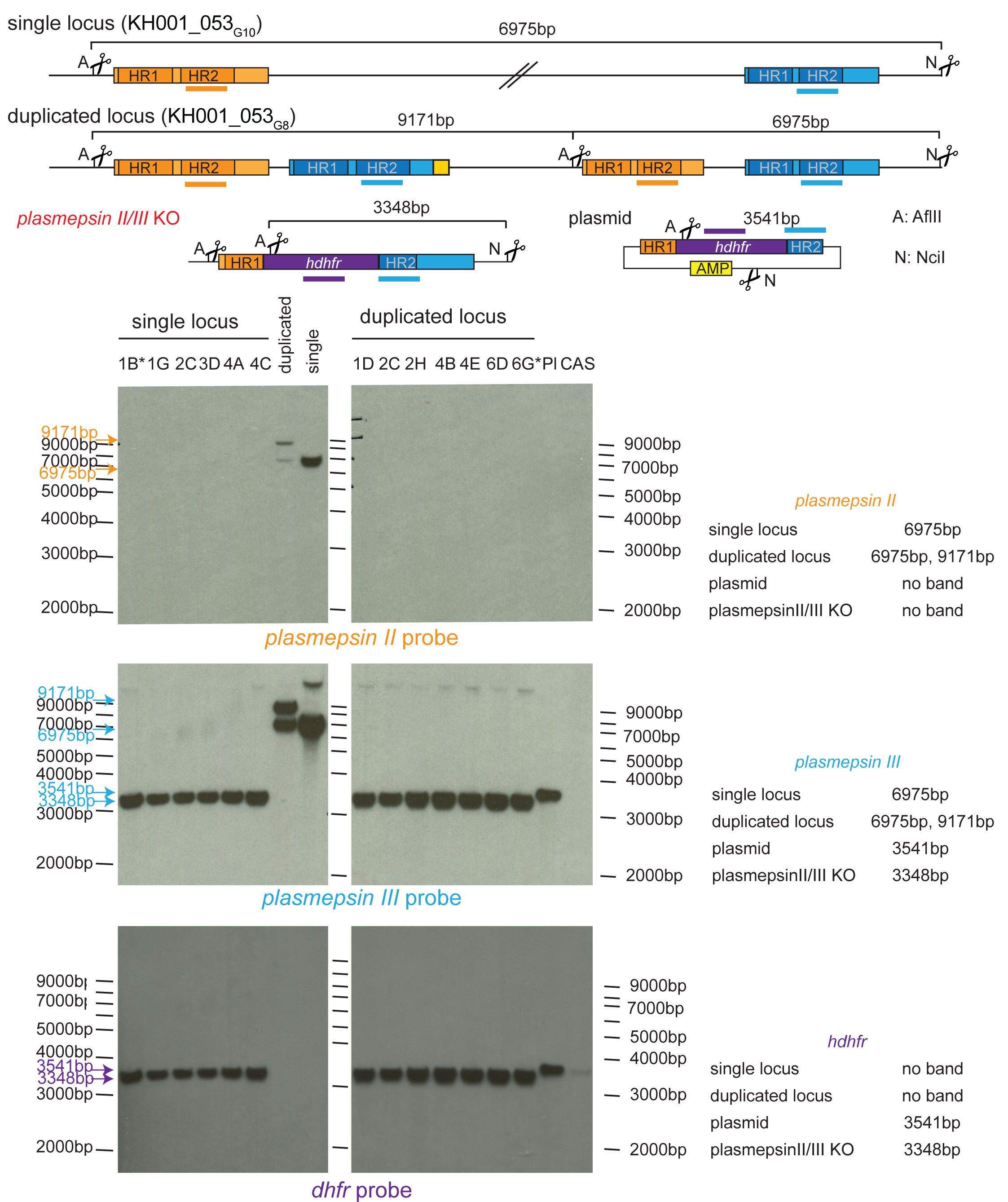

Supplement: S3 Fig — (A) Schema of original parental loci, homology plasmid used for integration into the loci and resulting edited locus which is identical for both parents. Restriction enzyme sites outside the locus were selected to confirm complete deletion of the regions between the homology regions and expected band sizes for Southern blots are indicated in the schema. Clones indicated with a star were used for phenotyping. (B) The same Southern blots was hybridized three times with the plasmepsin II, plasmepsin III, or hdhfr probe. Expected band size for each probe is indicated with arrows. The loss of hybridization for the plasmepsin II probe confirms the deletion and fusion of plasmepsin II and plasmepsin III in the KO clones. (TIF) [file ppat.1012779.s003.tif]

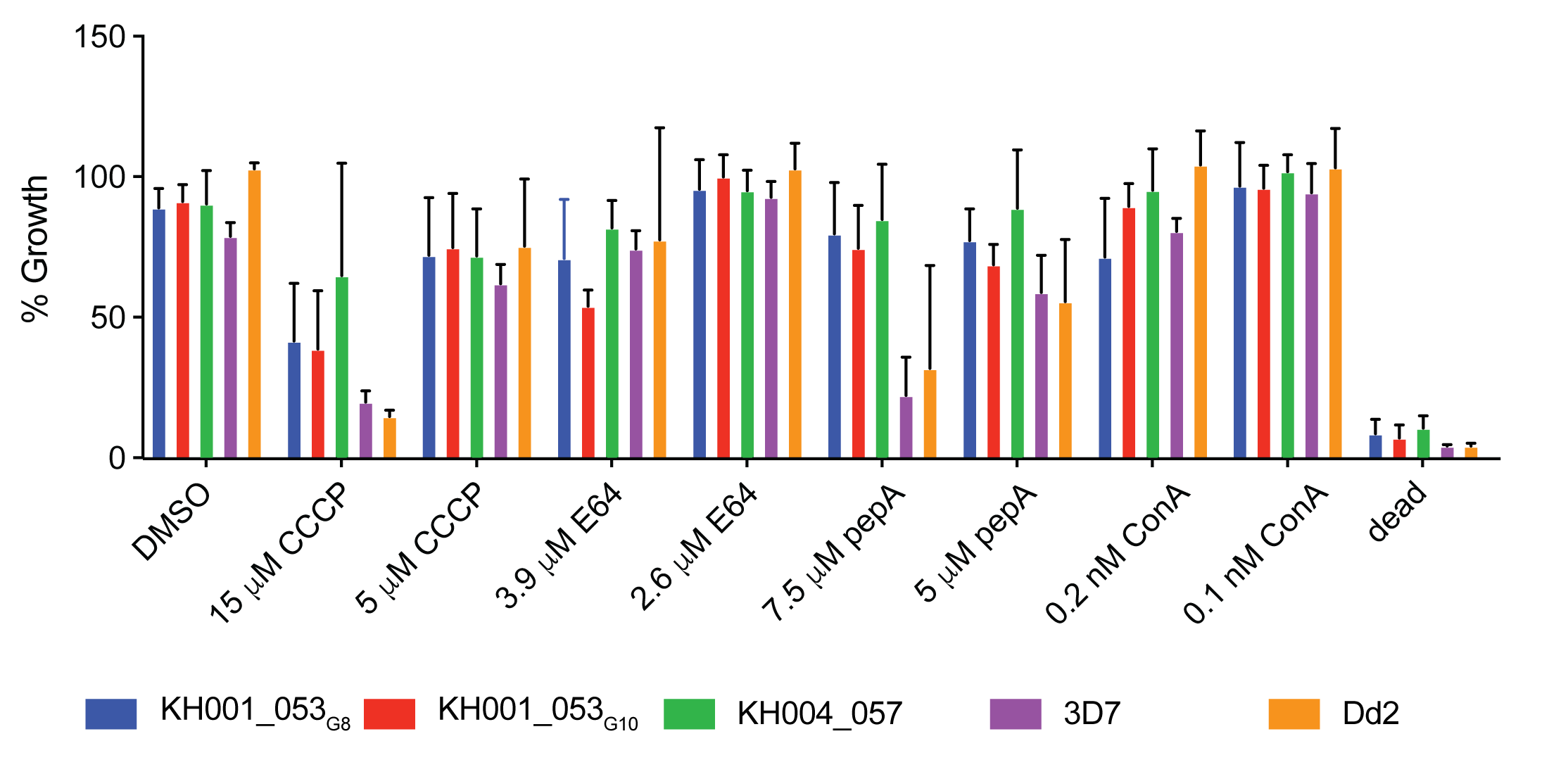

Supplement: S4 Fig — Ring-stage parasites were either grown in complete media or in complete media with the addition of CCCP (15 or 5 μM), E64 (3.9 or 2.6 μM), pepA (7.5 or 5 μM), ConA (0.1 or 0.2 nM), 10 μM DHA (dead) or 0.5% DMSO for 72 h. Growth was measured by the incorporation of SYBRGreen into DNA, read by a spectrometer and normalized to parasites cultured in media only. (TIF) [file ppat.1012779.s004.tif]

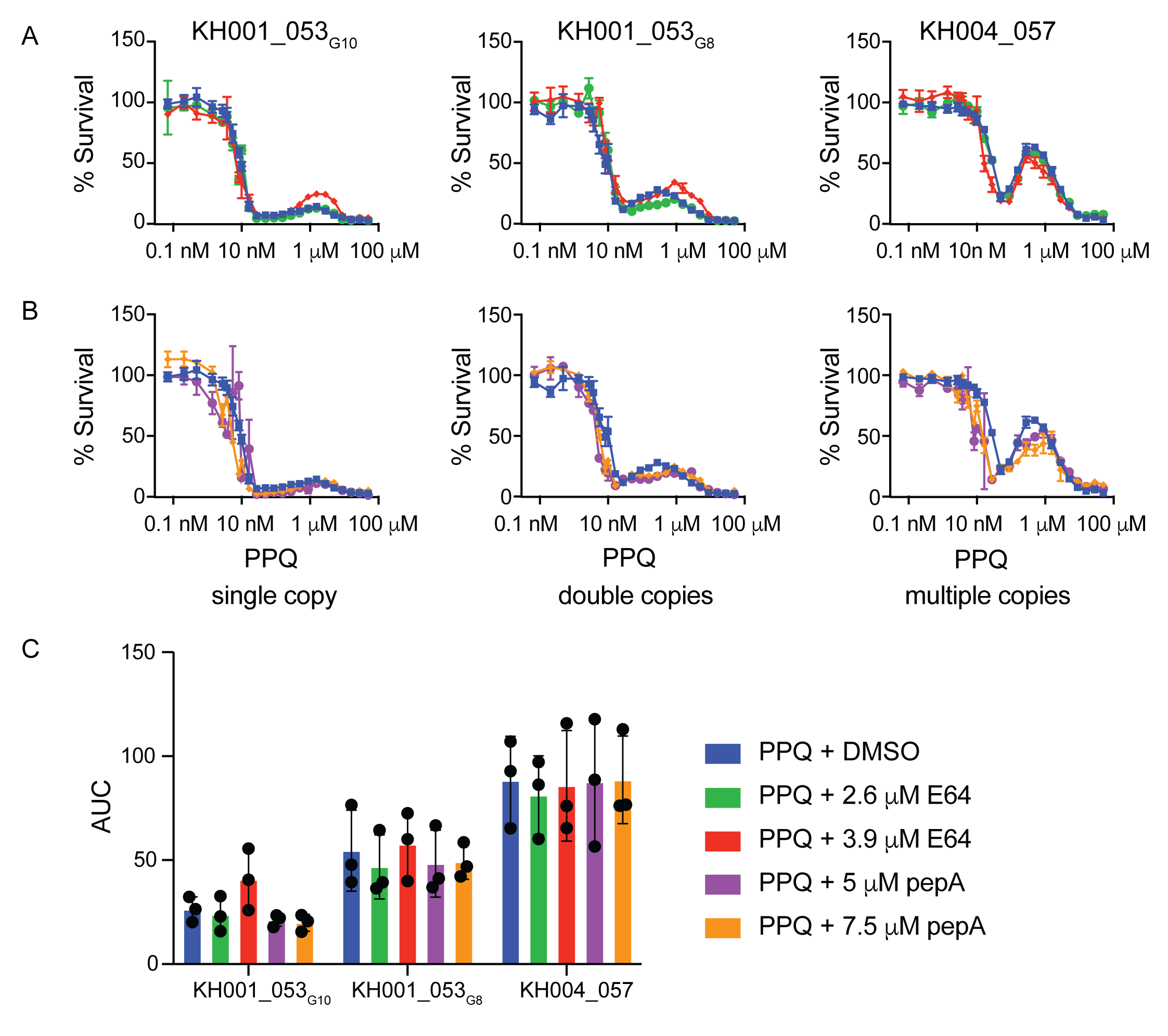

Supplement: S5 Fig — Parasites were exposed to increasing levels of PPQ in the presence of DMSO or (A) E64 at a concentration of either 2.6 μM or 3.9 μM or (B) pepstatin A (pepA) at a concentration of either 5 μM or 7.5 μM. Shown is one example of three biologically independent experiments run in triplicates. (C) Average and SD of the area under the curve (AUC) between the local minima for three biological replicates. No statistically significant difference was detected between PPQ alone and PPQ in combination with either E64 or pepA by ordinary one-way ANOVA with Tukey post-test. (TIF) [file ppat.1012779.s005.tif]

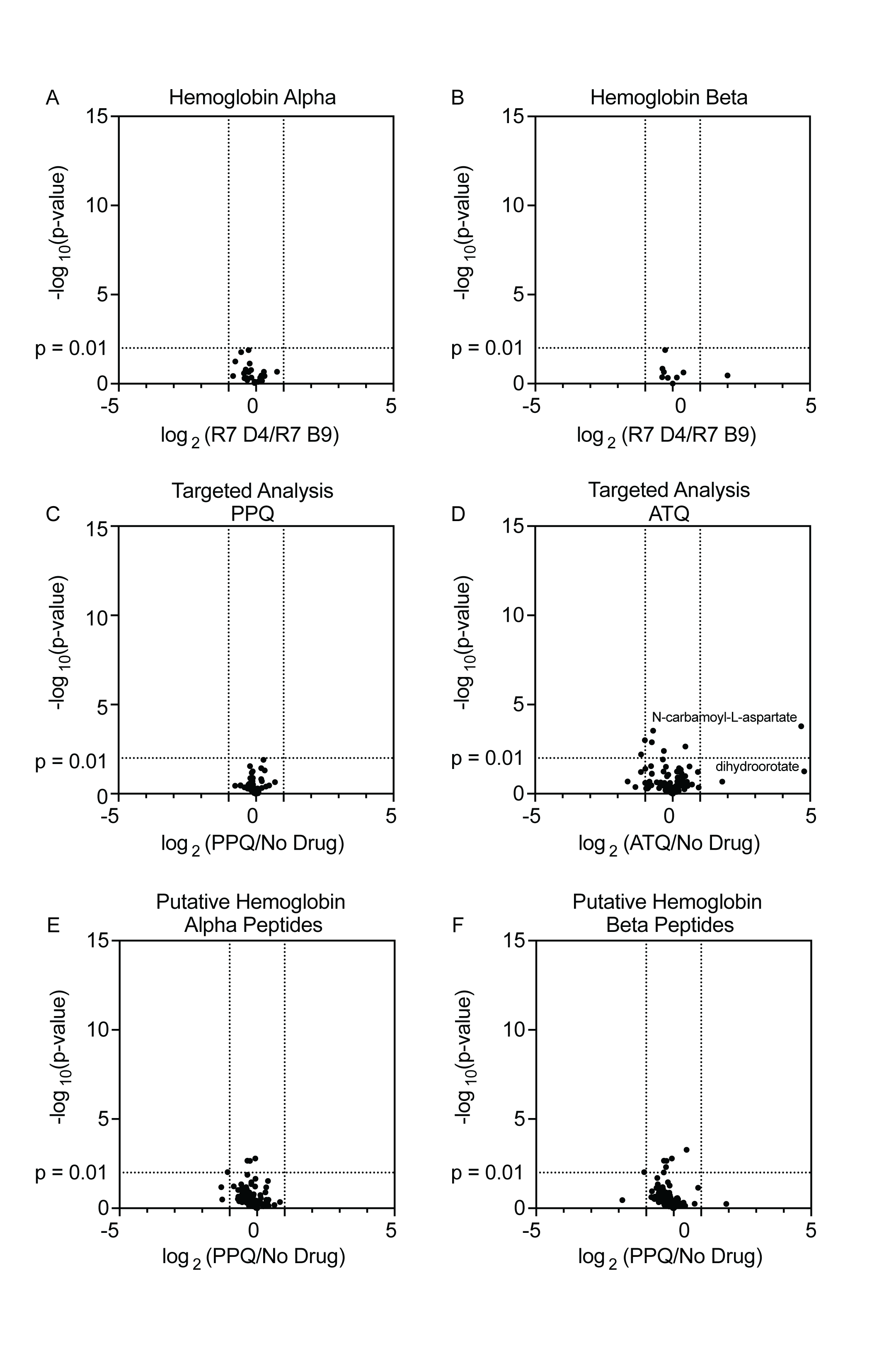

Supplement: S6 Fig — A and B: Two clones from the Cambodian RF7 parasite line [22] with either one copy (B9) or three copies (D4) of plasmepsin II and III were used for small molecule metabolomic analysis, which includes relative quantitation of short peptides. Metabolomic analysis was run in both positive and negative mode and a total of 35 putative endogenous hemoglobin-derived peptides (i.e., dipeptides to 13-mers) were detected based on their m/z match that could be mapped to either the alpha (A) or beta (B) chains of hemoglobin. Shown are the volcano plots combining statistical significance and fold change observed in metabolites from RF7 clones D4 compared to B9. C to F: Effects of PPQ or ATQ treatment on the parasite’s metabolism. Purified P. falciparum 3D7 trophozoites were treated for 2.5 h with 140 nM PPQ or 10 nM ATQ (as a control) and volcano plots comparing metabolites from untreated vs PPQ-treated parasites for targeted metabolite analysis from PPQ (C) and ATQ (D) treated parasites compared to untreated are shown. Volcano plots comparing metabolites from untargeted analysis of all putative hemoglobin-derived peptides of amino acid length 13 or less are shown in (E) (alpha chain) and (F) (beta chain). The dotted lines depict the significance cutoff of p = 0.01 and a two-fold change in metabolite abundance. Only in N-carbamoyl-L-aspartate and dihydroorotate under ATQ treatment were significantly increased in abundance [46]. (TIF) [file ppat.1012779.s006.tif]

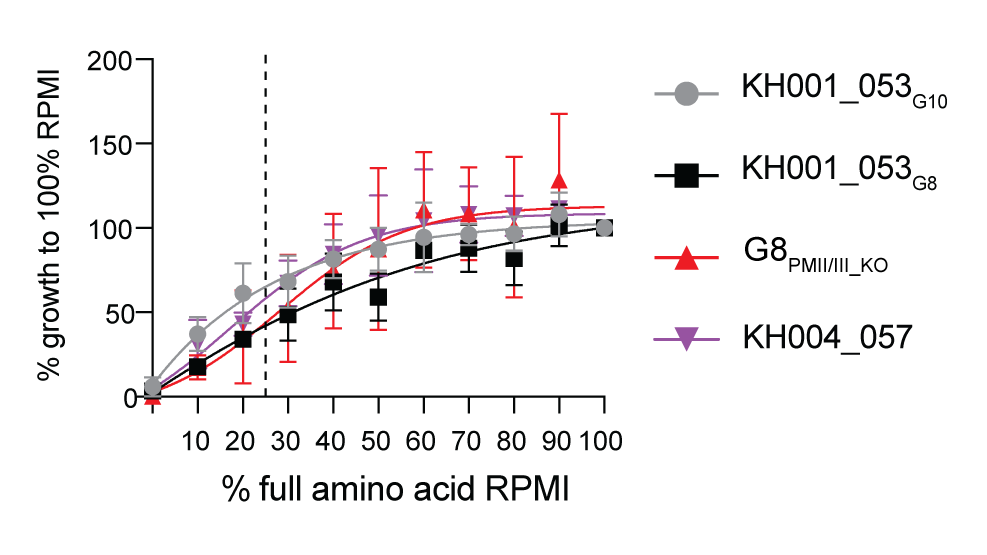

Supplement: S7 Fig — We determined the minimal amino acid needs of parasites to allow for enough DNA replication to perform drug susceptibility assays by SYBR Green I staining. KH001_053G10, KH001_053G8, G8PMII/III_KO and KH004_057 were synchronized and set up at 1% parasitemia and 2% hematocrit in regular RPMI media or RPMI media with isoleucine, methionine, and glutamine as the only amino acid sources. The two conditions were then mixed in 10% increments (90% regular media plus 10% amino acid-free (except isoleucine, methionine, and glutamine) media, 80% and 20% etc.) in 96 well plates and incubated at 37°C for 72 h. Growth was analyzed by adding SYBR Green to the plates, measuring the fluorescence, and normalizing the signal to parasites grown in regular media. The final assays conditions for drug susceptibility assays were set at 25% full amino acid RPMI and termed amino acid-limited media. (TIF) [file ppat.1012779.s007.tif]

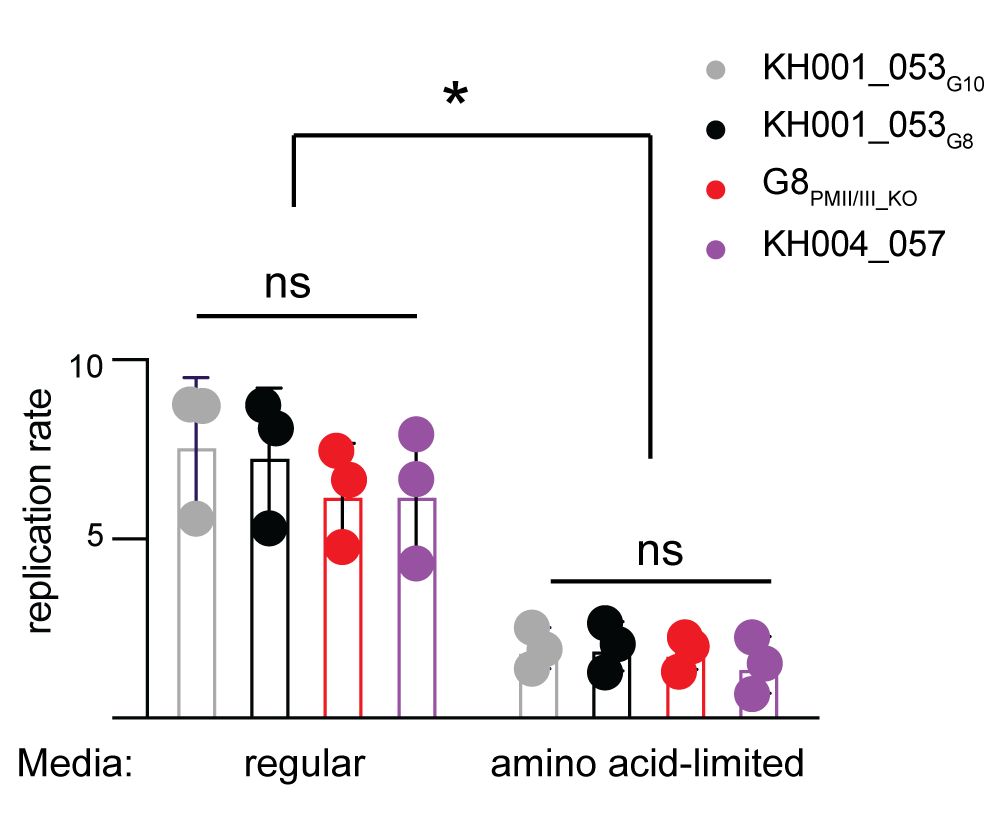

Supplement: S8 Fig — KH001_053G10, KH001_053G8, G8PMII/III_KO, and KH004_057 parasites were synchronized and set up at 0.5% parasitemia in either regular media or amino acid-limited media. Parasite replication was measured by estimating the parasitemia in the second cycle by flow cytometry of SYBR Green-stained parasite samples and dividing it by the initial parasitemia. Shown are the average replication rates for three biological replicates with SD. There were no statistically significant differences detected between the strain grown in either regular or amino acid-limited media by one-way ANOVA followed by Dunnett’s post-test, ns = no significance. The replication rate for all strains was significantly less in amino acid-limited media compared to regular media by Student’s t-test: *p < 0.05. (TIF) [file ppat.1012779.s008.tif]

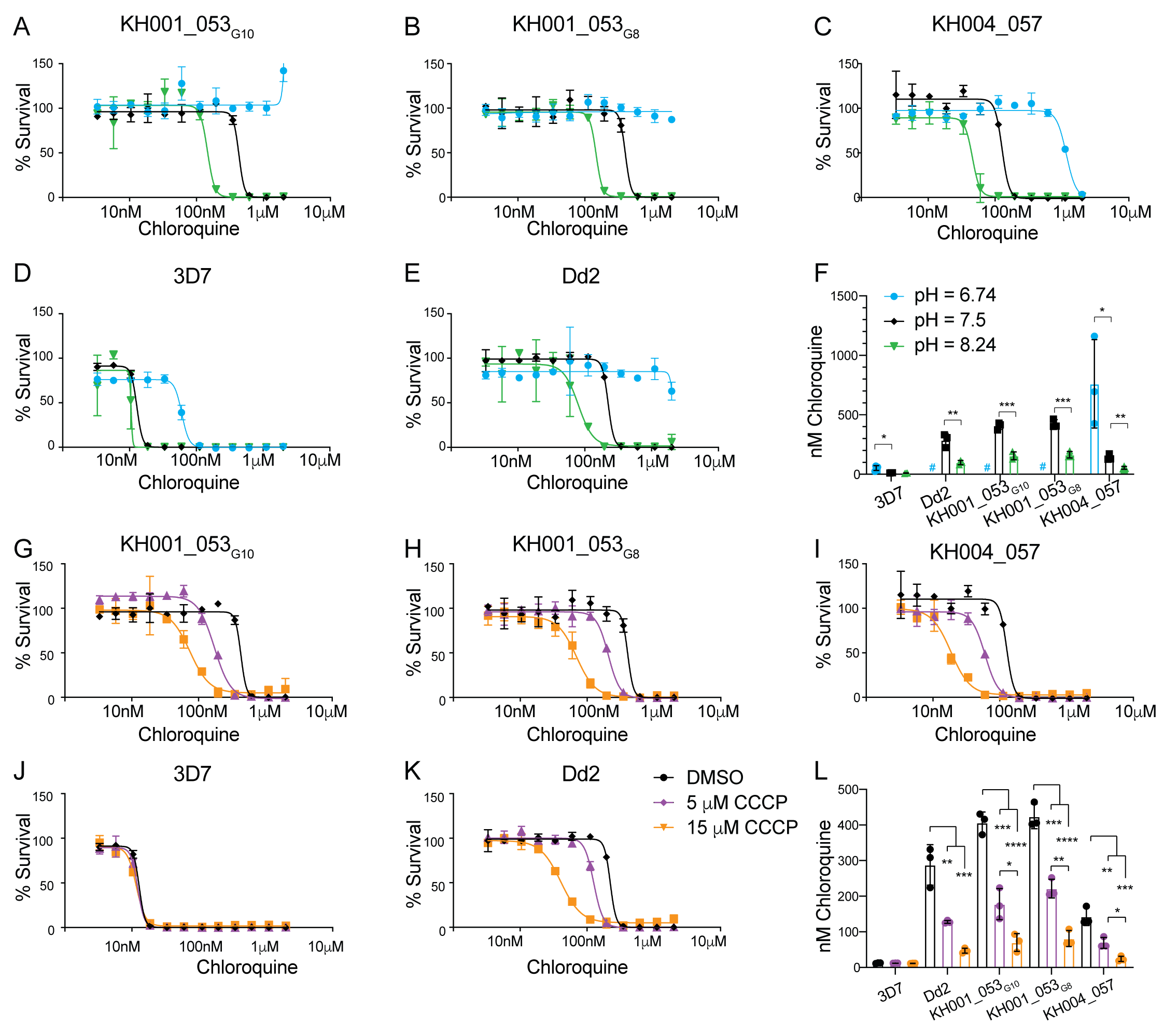

Supplement: S9 Fig — A-F) Parasites were exposed to increasing levels of CQ in acidic (pH = 6.74), normal (pH = 7.5) or basic (pH = 8.24) media. Shown is one example for each tested line of three biologically independent experiments run in triplicates. G-L) Parasites were exposed to increasing levels of CQ in the presence of DMSO or CCCP at a concentration of either 5 μM or 15 μM. The EC50 was calculated where possible (an # indicates when parasites were not killed completely at the highest concentration), and the average and SD are shown in (F and L). Statistics show one-way ANOVA with Tukey post-test for each strain tested in the presence of CCCP or two tailed paired Student’s t-test for the external pH changes: *p < 0.05; **p < 0.01; ***p < 0.001. (TIF) [file ppat.1012779.s009.tif]
